# Long‐term urbanization impacts the eastern golden frog (*Pelophylax plancyi*) in Shanghai City: Demographic history, genetic structure, and implications for amphibian conservation in intensively urbanizing environments

**DOI:** 10.1111/eva.13156

**Published:** 2020-11-07

**Authors:** Xu Wei, Meiling Huang, Qu Yue, Shuo Ma, Ben Li, Zhiqiang Mu, Chuan Peng, Wenxuan Gao, Wenli Liu, Jiaxin Zheng, Xiaodong Weng, Xiaohui Sun, Qingqiu Zuo, Shunqi Bo, Xiao Yuan, Wei Zhang, Gang Yang, Youzhong Ding, Xiaoming Wang, Tianhou Wang, Panyu Hua, Zhenghuan Wang

**Affiliations:** ^1^ School of Life Sciences East China Normal University Shanghai China; ^2^ Shanghai Landscaping & City Appearance Administrative Bureau Shanghai Forestry Bureau Shanghai China; ^3^ Natural History Research Centre of Shanghai Natural History Museum Shanghai Science and Technology Museum Shanghai China; ^4^ Shanghai Science and Technology Museum Shanghai China; ^5^ Institute of Eco‐Chongming Shanghai China; ^6^ School of Ecological and Environmental Sciences East China Normal University Shanghai China; ^7^ Joint Translational Science and Technology Research Institute East China Normal University Shanghai China; ^8^ Yangtze Delta Estuarine Wetland Ecosystem Observation and Research Station Ministry of Education & Shanghai Science and Technology Committee Shanghai China

**Keywords:** amphibian, conservation biology, *Pelophylax plancyi*, population genetics, Shanghai, urbanization

## Abstract

Understanding the mechanisms of how urbanization influences the evolution of native species is vital for urban wildlife ecology and conservation in the Anthropocene. With thousands of years of agriculture‐dominated historical urbanization followed by 40 years of intensive and rapid urbanization, Shanghai provides an ideal environment to study how the two‐stage urbanization process influences the evolution of indigenous wildlife, especially of anuran species. Therefore, in this study, we used mitochondrial *Cyt‐b* gene, microsatellite (SSR), and single nucleotide polymorphism (SNP) data to evaluate the demographic history and genetic structure of the eastern golden frog (*Pelophylax plancyi*), by sampling 407 individuals from 15 local populations across Shanghai, China. All local populations experienced bottlenecks during historical urbanization, while the local populations in urban areas maintained comparable contemporary effective population sizes (*N*_e_) and genetic diversity with suburban and rural populations. Nevertheless, the rapid modern urbanization has already imposed significant negative effects to the integrity of populations. The 15 local populations were differentiated into eight genetic clusters, showing a spatial distribution pattern consistent with the current urbanization gradient and island–mainland geography. Although moderate gene flow still occurred from the rural peripheral cluster to urban and suburban clusters, population fragmentation was more serious in the urban and suburban populations, where higher urbanization levels within 2‐km radius areas showed significant negative relationships to the *N*_e_ and genetic diversity of local populations. Therefore, to protect urban wildlife with limited dispersal ability, improving conditions in fragmented habitat remnants might be most essential for local populations living in more urbanized areas. Meanwhile, we highlight the need to preserve large unfragmented rural habitats and to construct corridor networks to connect discrete urban habitat remnants for the long‐term wildlife conservation in intensively urbanizing environments.

## 1 INTRODUCTION

Urbanization is developing rapidly across the world (Grimm et al., 2008; Liu et al., 2020; Seto et al., 2012). Habitat loss, fragmentation and isolation, and degradation caused by urbanization have become the most severe threats to wildlife (Grimm et al., 2008; McKinney, 2002). Amphibians are the most vulnerable taxonomic group of terrestrial vertebrates (Stuart et al., 2004), with 40% of species on the edge of extinction (IUCN, 2019). Their relatively limited dispersal ability (Smith & Green, 2005) and higher sensitivity to environmental changes (Cushman, 2006; Hamer & McDonnell, 2008) result in amphibians being impacted more by urbanization than other terrestrial vertebrates (Seto et al., 2012). In turn, the population status of amphibian species is frequently used as an indicator to evaluate the ecological impacts of urbanization (Guzy et al., 2012). Therefore, as reviewed by Lambert and Donihue (2020), knowledge about the response of amphibian populations to urbanization is not only essential to understand how anthropogenic activities affect the ecology and the evolution of amphibian species, but this information is also valuable for wildlife conservation in urbanized environments.

Understanding the population genetics of the target species and its relationship with environmental contributors is an effective methodology for studying urbanization impacts on wildlife. However, genetic responses to urbanization can be diverse among species, from significant population divergence to no obvious differentiation associated with urbanization (reviewed by Johnson & Munshi‐South, 2017; Miles et al., 2019). Even in the same species, conclusions from different studies about the urbanization effect can be inconsistent, even contradictory. For example, in studies of the wood frog (*Lithobates sylvaticus*) in Canada, Crosby et al. (2009) suggested that urban fragmentation in Ontario resulted in greater genetic differentiation and diversity loss in urban populations, whereas Furman et al. (2016) did not find significant genetic differentiation in Alberta. In addition to species attributes (Hamer & McDonnell, 2008), the mode of urbanization can also influence its impacts on the population structure. For example, in Oviedo, Spain, a city that is >1,300 years old, long‐term urbanization led to severe bottlenecks and significant population differentiation in the fire salamander (*Salamandra salamandra*; Lourenço et al., 2017). By contrast, in a rapid and pervasively urbanizing environment, such as New York City, fast divergence and significant loss of genetic diversity were observed in the northern dusky salamander (*Desmognathus fuscus*) within one century (Munshi‐South et al., 2013). Urban wildlife is usually influenced by temporal and spatial factors associated with urbanization simultaneously. Therefore, a specific area with a long history of human activity and rapid modern urbanization is an ideal research site to study population genetics of urban wildlife species.

According to the United Nations Population Division (2018), most of the growth in urbanization over the coming 30 years would be concentrated in developing countries. This explains why Hamer and McDonnell (2008) emphasized mitigating the geographic bias of current urban amphibian studies, which concentrate on relatively affluent regions (e.g., developed countries), by carrying out research in developing regions. As a typical example, urbanization in China is growing rapidly (Kuang et al., 2014; Shen et al., 2005), which provides ideal research sites to study wildlife adaptation during temporal and spatial continuums of urbanization development.

**Figure 1:**
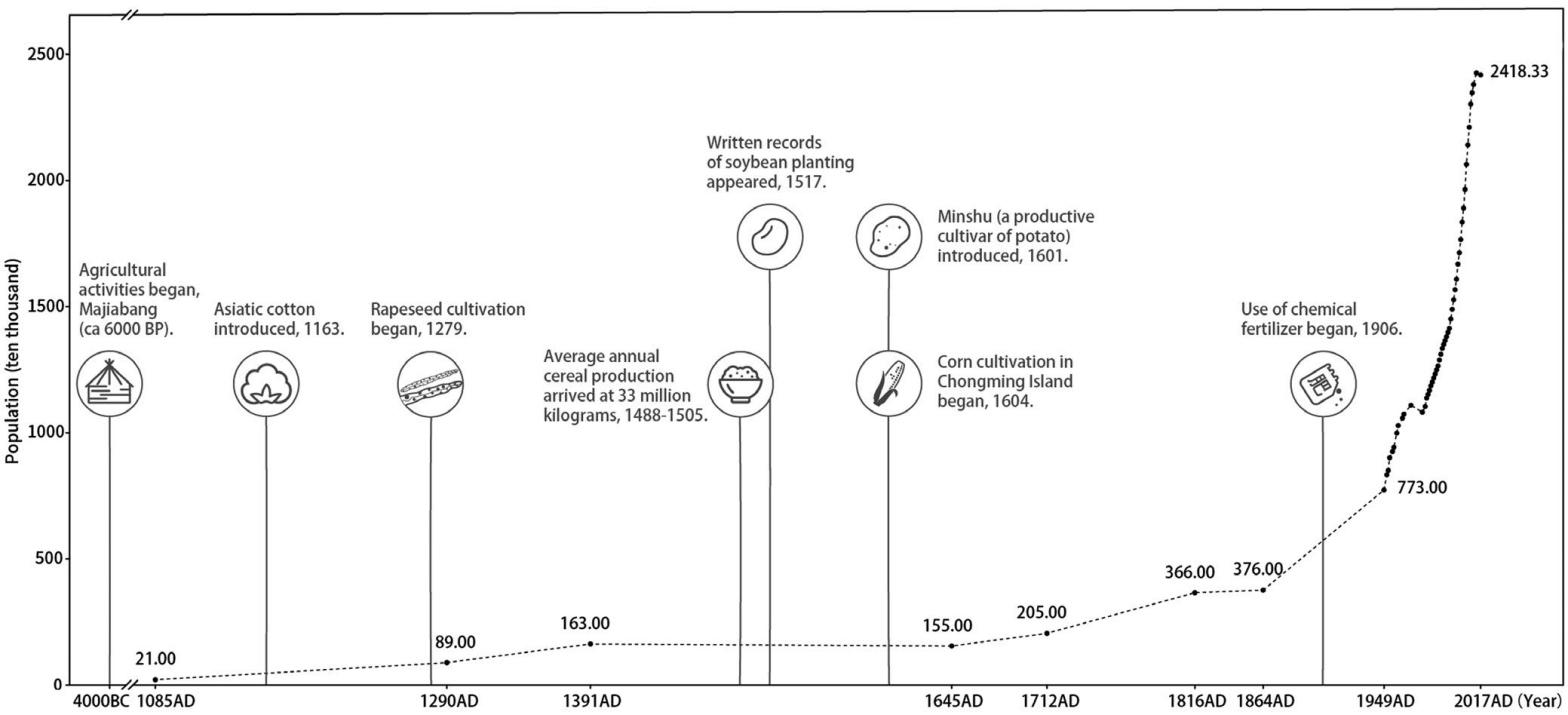
A brief history of demographic (dashed line, with a unit of 10,000 individuals) and city development records in Shanghai. Shanghai City experienced a period of slow population growth until the late 19th century (Hu, 1987). The growth rate began to increase during the 20th century. Particularly, over the past 40 years, the population has been explosively increasing (SMSB, 2018). The cultivation of rice already began in Majiabang (ca 6000 B.P.), and agriculture has continued to develop since then. Major agricultural events (Wang et al., 1996) related to important crops are indicated in the figure. Given the lack of detailed information from ancient time, the agricultural records shown are mainly within the past 1,000 years. Agricultural events are not shown during recent decades, because the conversion of land cover to impervious surfaces dominated during the modern rapid urbanization period (Tao et al., 2018)

**Figure 2:**
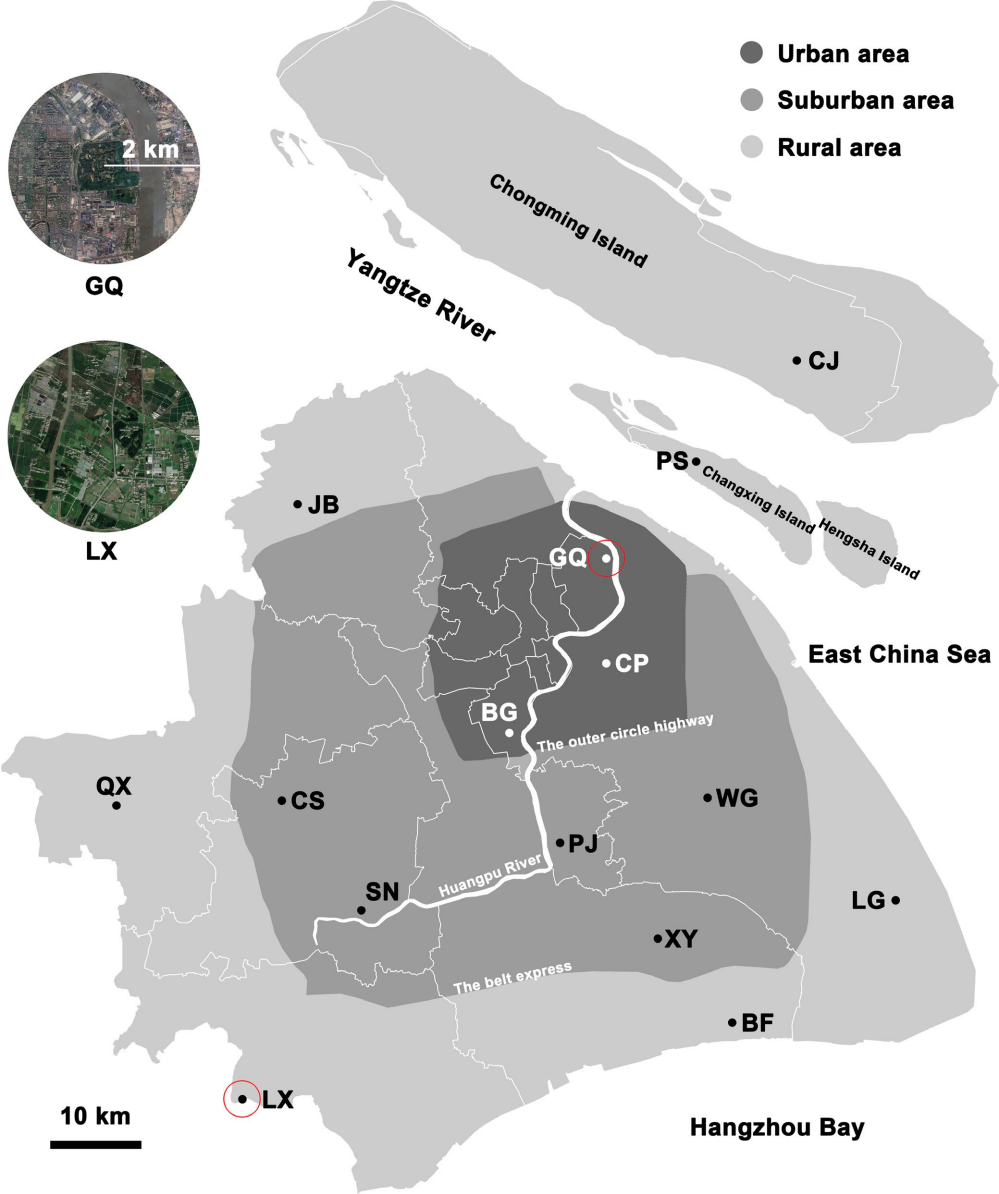
Distribution of the 15 *Pelophylax plancyi* sampling sites in Shanghai City. The boundaries of mainland areas with colors in different dark levels of gray represent the two main circular highways, the outer circle highway and the belt expressway. The insets show the local landscapes of two sampling sites within a 2‐km radius. LX is in the rural area with the lowest urbanization index (UI) of all 15 sampling sites, whereas GQ located in the urban area possesses the highest UI (see the details in Table 1). Site abbreviations correspond to those in Table 1

Urbanization is a complex process driven by the growth of human density, which causes severe landscape conversion from natural to human‐related types (Kinzig & Grove, 2001; McDonnell & Pickett, 1993), and it is linked with increased deforestation and agricultural intensification since the Iron Age (Boivin et al., 2016). As the third biggest megacity in the world (UNPD, 2018), Shanghai has been a major agricultural area in China since the 4th century B.C., and was constantly at the low‐level of historical urbanization dominated by agriculture until the late 19th century (Hu, 1987; Wang et al., 1996), when urbanization began to accelerate (Figure 1). Since Chinese economic reform started in 1978, Shanghai has been in a rapid and intensive modern urbanization period for >40 years, during which the resident population increased rapidly from 11 million (1978) to 24.18 million (2017; SMSB, 2018). Meanwhile, land use and land cover have been experiencing rapid and irreversible changes, from agricultural type to a range of impervious surface (Shi et al., 2018; Yin et al., 2011). The concentric‐ring expansion from the city center to the periphery (Kuang et al., 2014) established a complete urbanization gradient at the cityscape scale. Circular highways were constructed during 1993–2015, dividing Shanghai into urban, suburban, and rural areas (Figure 2). The urban area (within the outer circle highway, covering 664 km^2^) is most heavily urbanized, with 84.6% of the total built‐up area (Tao et al., 2018) and a human density of 23,841/km^2^ (SMSB, 2018). The suburban area, between the outer circle highway and the belt expressway, has been developing quickly since the 1990s, while the rural area outside of the belt expressway, including islands, remains dominated by agriculture ecosystem. Therefore, the long urbanization history and the rapid modern expansion of a concentric urbanization gradient render Shanghai as an ideal natural laboratory to study the impact of urbanization on wildlife in the city.

Synchronized with the modern urbanization, the amphibian biodiversity has decreased rapidly in Shanghai over the past 40 years. At least 11 anuran species were recorded living in the city until the 1980s (Huang et al., 1980), decreasing to eight by 2001 (SFB, 2004), and only five in 2015 (The Second National Terrestrial Vertebrates Survey, Shanghai, unpublished). Among the five anuran species, the eastern golden frog (*Pelophylax plancyi*) is most abundant and widely distributed across the city (Huang et al., 2018; Li et al., 2018; Zhang et al., 2016). As a typical pond‐breeder (Fei et al., 2010), *P. plancyi* may be the most sedentary anuran species in Shanghai, being observed mainly in or around water bodies (e.g., ponds, small rivers, and drainage ditches; Li et al., 2017; Yue, 2019). Amphibians, especially anuran species, usually breed in waters and wetlands, and live on land during the nonbreeding seasons. Therefore, the landscape complementation is required to complete the complex life cycles of amphibians (Pope et al., 2000). Meanwhile, urbanization characterized by habitat degradation and fragmentation is usually caused by impervious land surface changes like bridges and roads, which are highly risky to the survival of amphibians (Carr & Fahrig, 2001; McCartney‐Melstad et al., 2018). Thus, amphibian species as habitat generalists or with low dispersal requirements might survive better in urban environments (Hamer & McDonnell, 2008). However, with limited dispersal ability, the habitat attributes at the local scale can have a significant effect on the local populations. Previous research indicated that urban landscape configuration (Li et al., 2016, 2018; Zhang et al., 2016) and microhabitat characteristics (Huang et al., 2018; Yue, 2019) significantly impacted the local population sizes of *P. plancyi* in Shanghai. However, the current genetic variation in these local populations is unknown, and this knowledge is important for understanding the fate of small isolated populations and their persistence in these urban habitats. Moreover, given the long‐term development history of Shanghai City, we are interested in knowing how the urbanization process affects population characteristics of wildlife species. Such information is essential to understand the evolutionary processes affecting species in cities, to more effectively guide wildlife conservation in urban environments.

Therefore, to evaluate the influence of the long‐term urbanization on *P. plancyi* in Shanghai, we studied the population genetics of 407 individuals from 15 local populations across the entire urbanization gradient. We used mtDNA *Cyt‐b* gene to test whether the long‐term development history and natural barriers like big rivers and islands have caused subspecies differentiation. To study the genetic structure of local populations, microsatellite (SSR) and single nucleotide polymorphism (SNP) were the two kinds of most frequently used markers (see review by Miles et al., 2019). Although microsatellite loci usually have much higher mutation rates than that of SNP loci (Kruglyak et al., 1998; Martínez‐Arias et al., 2001), typically only a limited number of SSR loci were employed in each study, which may reduce sensitivity in detecting genetic diversity at genome‐wide levels (Väli et al., 2008). Consequently, the whole genome SNP techniques were employed increasingly in recent years (McCartney‐Melstad et al., 2018; Miles et al., 2019). However, SSR and SNP usually have different frequency distribution patterns as well as mutation rates and mechanisms (Morin et al., 2004). Therefore, both SSR and SNP were used in this study to give a more comprehensive evaluation of the genetic structure of *P. plancyi* local populations.

We hypothesized that the two‐stage urbanization process has imposed different effects on the *P. plancyi* population in Shanghai City. We predicted that the *P. plancyi* population experienced bottlenecks during the historical urbanization period because of the agriculture‐dominated interruption, while the explosive growth of human population and irreversible land‐cover conversion has imposed strong isolation effects on local populations in the 40‐year modern stage. Meanwhile, the genetic diversity of local populations located in more fragmented habitats in urban areas is lower than in more connected suburban and rural habitats. We also discussed implications of our results to the conservation biology in heavily urbanized megacities.

## 2 MATERIALS AND METHODS

### 2.1 Sampling sites and sample collection

Shanghai is located in the alluvial plain of the Yangtze River with a total area of 6,340 km^2^, comprising a mainland section and three main islands (Chongming, Changxing, and Hengsha). Huangpu River, the largest river in Shanghai, flows through the mainland area, dividing it into eastern and western parts. We collected 407 samples of *P. plancyi* across 15 sampling sites (Table S1) from May to August, 2017 and 2018, covering the urbanization gradient (i.e., urban, suburban, and rural; Figure 2). Ten of the 15 sites were parks or public woods, which were selected following two criteria: (a) an area of >10 ha; (b) built on former nursery gardens or agricultural landscape > 40 years ago, where the original water and drainage systems were largely retained. The other five sampling sites were all located in the remaining agricultural landscape in the suburban or rural areas, including Changxing and Chongming islands (Figure 2). Animals were captured by net or by hand. Toe‐clipped tissues were collected and stored in 75% ethanol. Animal handling procedures were approved by the Institutional Animal Care and Use Committee of East China Normal University (Protocol Number: LQ20190301).

### 2.2 Landscape variables

To study the impact of urbanization on *P. plancyi* population genetics, we collected landscape data at both the cityscape and local scales across Shanghai City. At the cityscape scale, using the two main circle highways as boundaries (i.e., the outer circle highway and the belt expressway), the 15 sampling sites were categorized into urban, suburban, and rural groups (Table 1) across the urbanization gradient (Figure 2). At the local scale, we followed the methods of Zhang et al. (2016). Landscape data within a 2‐km radius of the center of each sampling site were collected based on land‐cover data from satellite images of Formosat‐2 (June 2012; 2‐m resolution) and Google Earth Pro 7.3.2 (Google). Using ArcGIS v10.2 (ESRI), land‐use types, including woodlands, farmlands, grasslands, buildings, roads, and water bodies, were identified. Seven landscape variables were calculated by ArcGIS and Fragstats v4.2.1 (McGarigal et al., 2012): percentage of impervious surface (PIS), number of patches (NP), edge density (ED), landscape shape index (LSI), mean patch area (AREA), shape index (SHAPE), and Euclidean nearest‐neighbor distance (ENN).

**Table 1:** Population genetic statistics from 15 local populations of *Pelophylax plancyi* using three types of molecular markers

Local Population	Type	UI	mtDNA (*n* = 364)				SSR (*n* = 407)			SNP (*n* = 150)			
			*N*	*N*_H_	*h*	*π*	*N*	*N*_A_	*H*_O_	*N*	*P*_M_	*H*_O_	*π*
Century Park (CP)	U	0.577	31	9	0.714	0.004	32	7.769	0.620	10	0.832	0.205	0.248
Shanghai Botanical Garden (BG)	U	0.800	28	9	0.855	0.005	28	8.000	0.607	10	0.838	0.197	0.239
Gongqing Forest Park (GQ)	U	1.000	15	4	0.619	0.003	16	6.077	0.630	10	0.838	0.206	0.236
Chenshan Botanical Garden (CS)	S	0.669	34	7	0.629	0.002	36	7.769	0.588	10	0.843	0.206	0.230
Songnan Country Park (SN)	S	0.299	32	13	0.857	0.004	35	8.462	0.620	10	0.832	0.211	0.251
Pujiang Country Park (PJ)	S	0.109	12	6	0.803	0.003	15	7.077	0.605	10	0.837	0.202	0.245
Xiangyang Village (XY)	S	0.406	22	6	0.632	0.002	25	7.462	0.588	10	0.830	0.215	0.254
Wuzao Gang (WG)	S	0.202	33	7	0.583	0.002	34	8.231	0.559	10	0.821	0.301	0.268
Jiabei Country Park (JB)	R	0.072	13	8	0.897	0.004	17	7.692	0.597	10	0.830	0.214	0.254
Qingxi Country Park (QX)	R	0.087	30	13	0.874	0.003	33	8.231	0.601	10	0.833	0.210	0.250
Langxia Country Park (LX)	R	0.000	27	12	0.826	0.003	39	8.769	0.615	10	0.832	0.212	0.251
Bay Forest Park (BF)	R	0.623	11	5	0.618	0.002	11	6.462	0.622	10	0.833	0.213	0.249
Lingang Town (LG)	R	0.530	25	5	0.470	0.001	28	7.923	0.580	10	0.832	0.208	0.251
Panshi Town, Changxing Island (PS)	R	0.319	25	7	0.777	0.004	30	8.769	0.664	10	0.823	0.205	0.262
Chenjia Town, Chongming Island (CJ)	R	0.376	26	8	0.794	0.005	28	8.308	0.607	10	0.830	0.210	0.248
Local Scale	RC	—	—	−4.986	−0.213	0.000	—	−1.480	0.014	—	0.009	−0.022	−0.020
	$R_{\text{adj}}^{2}$	—	—	0.207	0.171	0.000	—	0.259	0.000	—	0.174	0.001	0.320
	*p*‐value	—	—	0.050*	0.070	0.962	—	0.031*	0.547	—	0.068	0.333	0.016*
Cityscape Scale	RC	−0.223	—	0.478	0.017	0.000	—	0.348	0.000	—	−0.003	0.001	0.005
	$R_{\text{adj}}^{2}$	0.321	—	0.000	0.000	0.000	—	0.062	0.000	—	0.084	0.000	0.126
	*p*‐value	0.016*	—	0.633	0.716	0.367	—	0.189	0.984	—	0.154	0.931	0.106

Note

Type—the relative location of the local population in the city (U – urban; S – Suburban; R – Rural); UI—urbanization index; *n*—total number of individuals genotyped by each type of molecular marker; *N*—number of samples genotyped in each local population using each type of marker; *N*_H_—the number of haplotypes; *h*—haplotype diversity; *π*—nucleotide diversity; *N*_A_—the number of mean alleles per loci; *H*_O_—observed heterozygosity; *P*_M_—mean frequency of the most frequent allele at each locus in each local population; the SNP‐derived genetic diversity indices were calculated based on the variable sites (SNPs themselves); Local scale—linear regression model (LRM) analysis results of genetic diversity versus UI at the local scale; Cityscape scale—LRM analysis results of genetic diversity versus urbanization gradient (1, 2, and 3 were assigned to urban, suburban, and rural, respectively) at the cityscape scale. RC—regression coefficient (LRM); $R_{\text{adj}}^{2}$—adjusted coefficient of determination.

* *p* < .05.

### 2.3 Double‐digest RADseq‐derived SNP data

Ten samples were randomly selected from each sampling site, so a total of 150 individuals were used for SNP data collection by double‐digest RADseq (ddRAD). Genomic DNA was extracted using the hexadecyltrimethylammonium bromide (CTAB) method. Double‐digest restriction‐associated DNA libraries were prepared using 500 ng of DNA per sample following the protocol described by Peterson et al. (2012) with some modifications. Briefly, genomic DNA was digested at 37°C for 5 hr using the restriction enzymes *EcoRI* and *MspI*, followed by the ligation of Illumina adapter sequences and unique 8‐bp barcodes that differed by at least three bases. Pools of 24 individuals were combined and run on agarose gel, where fragments of 300–500 bp were manually excised and purified using a Zymoclean Gel DNA recovery kit (Integrated Sciences, Chatswood, Australia). Each pool was amplified using 14 PCR cycles in 25 μl reactions containing Phusion High‐fidelity PCR reagents, library DNA, and a unique indexing primer for each pool following the standard Illumina multiplexed sequencing protocol. DNA libraries were quantified using the high‐sensitivity DNA analysis kit in a 2100 Bioanalyser (Agilent Technologies). Pools were combined in equimolar concentration and sequenced in Illumina HiSeq 2500 2 × 150 bp pair‐end sequencing by Personal, Shanghai, China.

FastQC v0.11.7 (Andrews, 2010) was used for raw data quality control. Adapters and low‐quality sequences were removed, and reads < 50 bp were filtered out to obtain high‐quality reads, which had average lengths of ~138 bp. Sequences were all trimmed to 130 bp and were designed for SNP genotyping using Stacks v1.48 (Catchen et al., 2013). After several trial runs for parameterization, SNPs were genotyped in all 150 individuals by setting the minimum depth of coverage (*m*) to four when creating a stack, and setting the maximum distance (in nucleotides) between stacks (*M*) to two in *ustacks*. The number of mismatches allowed between sample loci (*n*) was 2 when building a catalog in *cstacks*. In the *populations* module, retained SNP loci had to be present in >75% (*r*) of the 150 individuals, and in at least 12 (*p*) of the 15 local populations with a minimum minor allele frequency (*min_maf*) of 0.05. In total, 71,910 SNP loci were obtained using the above parameters. An extra parameter (*write_single_snp*) was added to retain only the first SNP locus of each read; thus, a 44,469 SNP loci subset was kept to avoid linkage disequilibrium in the population structure analyses. To avoid the impacts of missing data and compromise with the computation limitation of the program, another subset with 1,611 SNP loci without missing alleles was obtained for gene flow analyses.

### 2.4 mtDNA amplification

Genomic DNA was extracted using a Genomic DNA Tissue Kit (Tiangen Biotech) from all 407 *P. plancyi* samples in the laboratory. The quantity and quality of extracted DNA were verified by electrophoresis in 1% agarose gel. All individuals were sequenced at a partial fragment of the *Cyt‐b* gene using the primers (B104F and B829R) and the PCR conditions described by Liu et al. (2010). PCR products were sequenced in both directions on an ABI3500 analyzer by Sangon Biotech, Shanghai, China. Sequences with poor sequencing quality were discarded, resulting in 364 individual sequences were retained for further analysis.

### 2.5 SSR locus selection and amplification

We selected SSR loci from previously published papers on *P. plancyi* (Dai & Zhou, 2009), and the closely related species *P. nigromaculatus* (Du et al., 2012) and *P. hubeiensis* (Yan et al., 2011). In total, 40 loci were selected from the three papers. However, many of them either failed to amplify, were nonspecifically amplified, or were monomorphic. Only 11 loci were kept for the following analyses. To acquire more genetic information from the SSR data, we developed four novel SSR loci from the RADseq data (see details in Appendix S1).

All 407 individuals were genotyped at the 15 SSR loci. GENEMAPPER v4.0 (Applied Biosystems) was used to perform allele scoring. We randomly selected 10% of all samples to carry out a second amplification to confirm the accuracy of genotyping. The primer information and thermal cycling details are listed in Table S2. MICRO‐CHECKER v2.2.3 (Oosterhout et al., 2004) was used to check allele dropout and null alleles. Deviations from Hardy–Weinberg equilibrium (HWE) and linkage equilibrium (LE) were tested in Genepop v4.6 (Rousset, 2008). The sequential Bonferroni adjustment (Rice, 1989) was used to correct *p* values from the HWE and LE multiple exact test. MICRO‐CHECKER showed no consistent signs of allele dropout or null alleles among the 15 local populations. A few loci deviated from HWE or LE in only one or two local populations, whereas two (Pn214 and Pla96) showed deviation from HWE in more than half of the 15 local populations, and thus, were deleted; 13 SSR loci were retained for further analysis.

### 2.6 Demographic history

The R package *VarEff* v1.2 (Nikolic & Chevalet, 2014) was employed to estimate the effective population size with a coalescent approach from the present to ancestral time using 13 SSR loci in each local population. We set 5,000 generations to cover a 5,000‐year history by assuming at least 1 year for *P. plancyi* to reach sexual maturity. Given that no research has been done in the mutation rate calculation in *P. plancyi* and its closely related species, we set the mutation rate to 0.00127, which was calculated in eastern tiger salamanders (*Ambystoma tigrinum*; Bulut et al., 2009). The two‐phased mutation model (*T*) was used with an additional coefficient (*C*) of 0.22, as recommended by Peery et al. (2012). For each local population, three replicates of simulations were carried out. To each simulation, we used 100,000 batches with a length of 10, thinned every 10 batches in the MCMC chain and with a burn‐in period of 10,000 batches. The other parameter settings were listed in Table S3. The effective population size value was output for each generation. The convergence of the simulation results was assessed by the Gelman‐Rubin test (Gelman & Rubin, 1992) which was conducted using the R package *coda* (Plummer et al., 2006). The time of bottleneck beginning (*T*_bot_) and of the strongest bottleneck effect (*T*_MG_) were evaluated in each local population. For *T*_bot_, a bottleneck was considered to start once there had been a 5% loss of the initial effective population size. *T*_MG_ was the time when the bottleneck showed the strongest effect and caused the fastest declining rate in each local population.

For SNPs, DIYABC v2.1.0 (Cornuet et al., 2014) was used to infer the demographic history in each local population using the approximate Bayesian computation method. We referred to three possible scenarios described by Low et al. (2018): population expansion, population recovery, and population contraction. A subset of 5,000 SNPs was randomly selected for this analysis. We set uniform prior distributions with the following ranges: 10 ≤ *T*_anc_ ≤ 5,000; 10 ≤ *T*_bot_ ≤ 500; 1,000 ≤ *N*_anc_ ≤ 5,000; and 10 ≤ *N*_bot_ ≤ 1,000; contemporary effective population size (*N*_e_) was set with default parameters. We generated 1 × 10^6^ simulations for each scenario. Three single‐population summary statistics for the 5,000 randomly selected SNP loci were chosen to perform simulations: (a) mean and (b) variance of gene diversity across polymorphic loci, and (c) mean gene diversity across all loci. Using a polychotomous logistic regression, the optimal scenario was chosen with the highest posterior probability value calculated with 1% of simulated data sets closest to the observed data sets. We then selected the 1,000 closest‐to‐observed pseudo‐observed data sets (PODs) to evaluate the posterior predictive error rate for the optimal scenario. Posterior parameter estimation was performed for the optimal scenario on the 1% of closest‐to‐observed PODs. Using the *Model Check* option, we ran the principal component analysis (PCA) to assess the goodness of fit of the optimal scenario from the set of 10,000 PODs simulated from the posterior predictive parameter distributions. For quantifying confidence of parameter estimations, the bias and precision of parameter estimates were assessed with 1% PODs drawn from posterior distributions.

### 2.7 Genetic diversity in local populations

For *Cyt‐b*, we used ARLEQUIN v3.5 (Excoffier et al., 2005) to calculate the number of haplotypes (*N*_H_), haplotype diversity (*h*), and nucleotide diversity (*π*) for each local population. For SSRs, GenAlEx v6.503 (Peakall & Smouse, 2012) was used to estimate the mean number of alleles per marker (*N*_A_) and the observed heterozygosity (*H*_O_). For SNPs, the *populations* module in Stacks was used to calculate *H*_O_, *π*, and the mean frequency of the most frequent allele at each locus (*P*_M_) in each local population concerning only the variant sites across the reads (i.e., SNPs themselves).

### 2.8 Genetic differentiation between local populations

For SSRs, we used ARLEQUIN to calculate *F*_ST_ between local populations. For SNPs, vcftools v0.1.17 (Danecek et al., 2011) was employed for *F*_ST_ estimation. The contribution of isolation by distance to genetic differentiation was evaluated by the Mantel test in GenAlEx.

### 2.9 Phylogenetic analysis of mtDNA

The *Cyt‐b* sequences of *P. bergeri* and *P. ridibundus* were downloaded from GenBank as out‐groups (accession ID: MF094318 and AB980792) and aligned with *P. plancyi* sequences using MEGA v7 (Kumar et al., 2016). DNASP v6.0 (Rozas et al., 2017) was employed to identify haplotypes, which were then imported into DAMBE v6 (Xia, 2017) for nucleotide substitution saturation tests. jModelTest v2.1.4 (Darriba et al., 2012) showed that the HKY + G model was the optimal substitution model for both maximum‐likelihood (ML) and Bayesian (BI) phylogenetic analyses. A ML tree was constructed by MEGA with 1,000 bootstrap replications. MrBayes v3.2.6 (Ronquist et al., 2012) was used to construct BI trees, using MCMC posterior probability estimations for 1.8 × 10^6^ generations with a 1,000‐generation sampling interval, discarding the first 25% aging samples when summing up. We used FigTree v1.4.3 (http://tree.bio.ed.ac.uk/software/figtree) to present the best BI tree. TCS v1.21 (Clement et al., 2000) was used to draw the haplotype network.

### 2.10 Population structure based on SSR and SNP data

For SSRs, STRUCTURE v2.3.4 (Pritchard et al., 2000) was used by setting 10 replicate runs for a series of clusters (*K*) from 1 to 15 with 1 × 10^6^ burn‐in and 2 × 10^6^ MCMC iterations for each run. The optimal *K* value was derived by the *ΔK* statistic (Evanno et al., 2005). CLUMPP v1.1.2 (Jakobsson & Rosenberg, 2007) was used to summarize and align the cluster membership coefficients from multiple independent runs. The results were visualized using District v1.1 (Rosenberg, 2004).

The R package *Geneland* v4.0.8 (Guillot et al., 2005) was employed using the correlated allele frequency model with prior information of spatial coordinates of each local population, and 1 × 10^6^ MCMC iterations were set with 100 as the thinning value for each *K* value. Ten replications were simulated, and the optimal *K* returned by the simulation with the highest average posterior probability was chosen for visualization. The discriminant analysis of principal components (DAPC; Jombart et al., 2010) in the R package *adegenet* v2.1.1 (Jombart, 2008) was used to reflect the extent of genetic differentiation among local populations using a nongenetic‐based model. The number of principal components (PCs) was determined by the *α‐*score cross‐validation function to avoid overfitting.

For SNPs, we used the 44,469 loci subset to perform genetic‐based model tests including STRUCUTRE and Admixture operations, and used all 71,910 loci for the DAPC analysis. We set five replicate runs in STRUCTURE for each *K* with a burn‐in of 1 × 10^4^ iterations and 2 × 10^4^ MCMC iterations. All the other settings in CLUMPP and District were the same as the SSR data analysis. Meanwhile, Admixture v1.3.0 (Alexander et al., 2009) was used to perform ML estimates using 2 × 10^4^ bootstraps. The optimal *K* value was calculated by the cross‐validation method (Alexander & Lange, 2011). DAPC analysis was also performed on the total 71,910 SNP loci following the same protocol as used for the SSR analysis.

The number of genetic clusters of the 15 local populations was determined by the consistent results of optimal *K* values from both SSR and SNP analyses.

### 2.11 Gene flow between genetic clusters

The 15 local populations were grouped into eight genetic clusters; eight was the optimal *K* value for SSR (*Geneland*) and SNP (STRUCTURE) analyses (see details in *Results* part). Using BayesAss3 (Wilson & Rannala, 2003), we tested recent migration rates between clusters using the 13 SSR loci and the subset of 1,611 SNP loci. All three mixing parameters were set to 1.0, with the acceptance rates maintained between 20% and 60%. The MCMC iterations were set to 1 × 10^7^ with a sampling interval of 100, and the first 1 × 10^6^ iterations were discarded as burn‐in. We ran three replicate analyses with different seed values on each type of marker. Convergence was visually assessed by Tracer v1.7 (Rambaut et al., 2018).

### 2.12 Urbanization index and statistical analysis

Referring to Zhang et al. (2016), we used PCA to integrate the seven landscape variables. The first PC (PC1) accounted for 70.6% of the total variance in the data (Table S4). Thus, the score of PC1 from each local population was scaled to a value between 0 and 1 as the urbanization index (UI), which was used to measure the urbanization level at the local scale. A higher UI represented a higher urbanization level of the sampling site habitat area within a 2‐km radius. A one‐sample *t* test was used to evaluate the significance of the UI value difference among local populations.

To test whether the local scaled UI showed a consistent trend with the cityscape urbanization gradient, we used a linear regression model (LRM) to test the relationship between UI and the urbanization gradient where the sampling site was located. To do so, we assigned values (*G*_A_) to the urbanization gradient as 1 for urban, 2 for suburban, and 3 for rural.

Moreover, LRMs were used to evaluate the relationships between genetic variation and the urbanization level at both the cityscape (*G*_A_) and local (UI) scales. The genetic variation indices of each local population as dependent variables involved in LRMs included all genetic diversity indices and contemporary effective population sizes (*N*_e_) calculated by SSRs and SNPs, and the urbanization level was the independent variable. Given that all the genetic variation indices tend to be correlated, and we wanted to test their relative priority of reaction to the urbanization, only one index in each LRM simulation was tested.

All statistical analyses were performed using R v3.5.1 (http://www.r‐project.org/), and the power of LRMs was evaluated by adjusted *R* ^2^.

## 3 RESULTS

### 3.1 Demographic history

**Figure 3:**
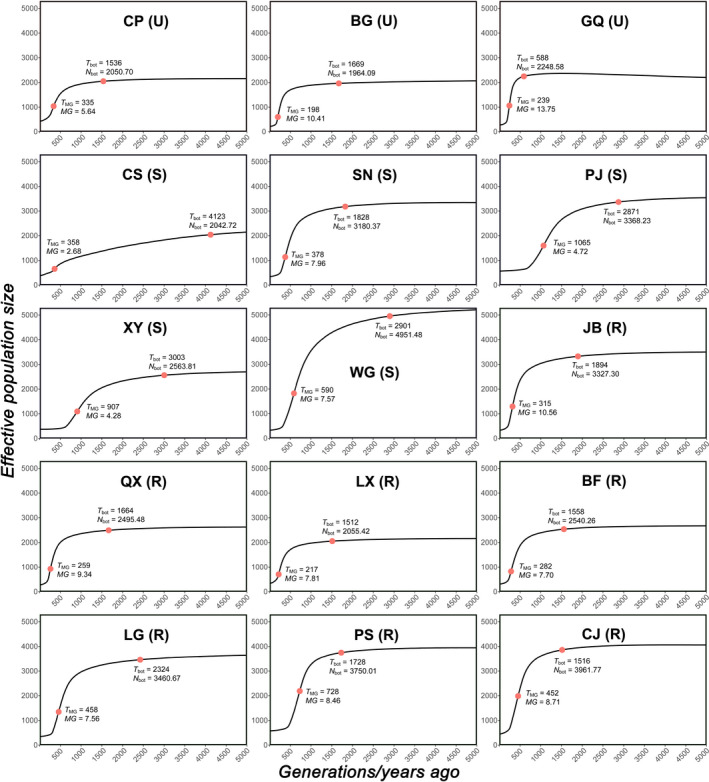
Demographic history of *Pelophylax plancyi* from the present to 5,000 generations/years ago in the 15 local populations calculated by *VarEff* using the 13 SSR loci. The abbreviation in parentheses represents the relative location of the local population in the city (U—Urban; S—Suburban; R—Rural). Red dots on each curve represent time points of the beginning (*T*_bot_) and the strongest effect (*T*_MG_) of bottleneck in each local population. Site abbreviations correspond to those in Table 1

The analysis using *VarEff* showed that all 15 local populations had experienced bottlenecks since historical urbanization (Figure 3). The Gelman‐Rubin test showed that the values of the potential scale reduction factor (PSRF) for almost all local populations were lower than 1.1, with only the value in QX (PSRF = 1.12) deviating slightly more, consistent with valid results by *VarEff* analyses (Table S5). The ancestral maximum effective population sizes (*N*_max_) ranged from 2,067.5 to 5,212.2, while the contemporary effective population sizes (*N*_e_) decreased to a range from 218.6 to 569.4 (Table S5). In most sampling sites, bottlenecks had already begun to affect local populations from at least 1,512 generations/years ago (*T*_bot_), except for GQ, where the bottleneck started 588 generations/years ago (Figure 3 and Table S5). The mean time of the strongest bottleneck effects (*T*_MG_) of all local populations was 452 ± 261.3 (mean ± *SD*; range, 198–1,065) generations/years ago. The maximum magnitude of bottleneck effect (*MG*) showed a moderate decrease of 7.8 ± 2.8 (2.7–13.8) individuals per generation/year (Table S5).

Coalescent modeling in DIYABC suggested optimal demography models of either population recovery or population expansion in all local populations with low posterior predictive error rates ranging from 0 to 0.286 (Table S6). Except for PS, the other 14 local populations were most likely to be experiencing a population recovery process from their big ancestral populations (*N*_anc_; 5,550 ± 894.3; 4,430–8,320) to the smallest (*N*_bot_; 262.1 ± 164.2; 37–509) through bottleneck effects that happened almost 400 years ago, except for 73.4 years ago in CS (356.4 ± 83.5; 73.4–410) and followed by recovering *N*_e_ (1,114.6 ± 736.0, 177–2,240; Table S7). For PS, a population expansion model was best suited with *N*_anc_ = 272, *T*_anc_ = 2,960, *N*_bot_ = 115, *T*_bot_ = 441, and *N*_e_ = 442 (Table S7). The goodness‐of‐fit test (Figure S1) showed that the observed data points were located in the clusters of both prior and posterior predictive distribution, indicating good model performances. Bias and error estimates for each posterior parameter of the optimal scenarios were generally small and were shown in Table S8.

### 3.2 Genetic diversity in local populations

In general, the genetic diversity levels in the 15 local populations were similar based on the analyses of the three types of molecular markers (Table 1). For mtDNA, the number of haplotypes (*N*_H_) of local populations ranged from 4 to 13. Most of the local populations had haplotype diversity (*h*) that ranged from 0.583 to 0.897, although LG showed the lowest value of 0.470 (Table 1). For SSRs, the mean number of alleles per locus (*N*_A_) ranged from 6.077 to 8.769. The observed heterozygosity (*H*_O_) ranged from 0.559 to 0.664 (Table 1). For SNPs, the mean frequency of the most frequent allele (*P*_M_) ranged from 0.821 to 0.843; *H*_O_ was similar, at ~0.214 (± 0.024) with the highest detected in WG (0.301) and the lowest in BG (0.197); and *π* ranged from 0.230 to 0.268 (Table 1).

### 3.3 Genetic differentiation between local populations

Pairwise *F*_ST_ values of SSRs and SNPs identified that the local populations within or near to urban areas (CP, BG, GQ and CS) and in the two islands (PS and CJ) showed higher differentiation than the other local populations (Table S9). The *F*_ST_ values related to these six local populations ranged from 0.026 to 0.107 in SNPs and from 0.017 to 0.078 in SSRs. Local populations in the urban area showed stronger differentiation than those on the islands to other local populations. For example, *F*_ST_ values between the urban GQ population and other local populations were the highest compared with *F*_ST_ values of the other 14 local populations (Table S9). Neither the SSR data nor SNP data revealed significant signal of isolation by distance by Mantel tests (*R* ^2^ = 0.030, *p = *.152 for SSRs; *R* ^2^ = 0.005, *p* = .345 for SNPs).

### 3.4 Phylogenetic analysis of mtDNA

In total, 68 haplotypes of a 656‐bp *Cyt‐b* fragment were obtained from 364 individuals. Among these haplotypes, H1 and H3 were the two main haplotypes, with 53 and 151 individuals, respectively (Figure S2). Both maximum‐likelihood (Figure S3) and Bayesian (Figure S4) trees showed consistent topological relationships between haplotypes, and genetic distances between haplotypes were small, thus indicating no significant divergence among the 15 local populations at the mitochondrial DNA level.

### 3.5 Population structure based on SSR and SNP data

**Figure 4:**
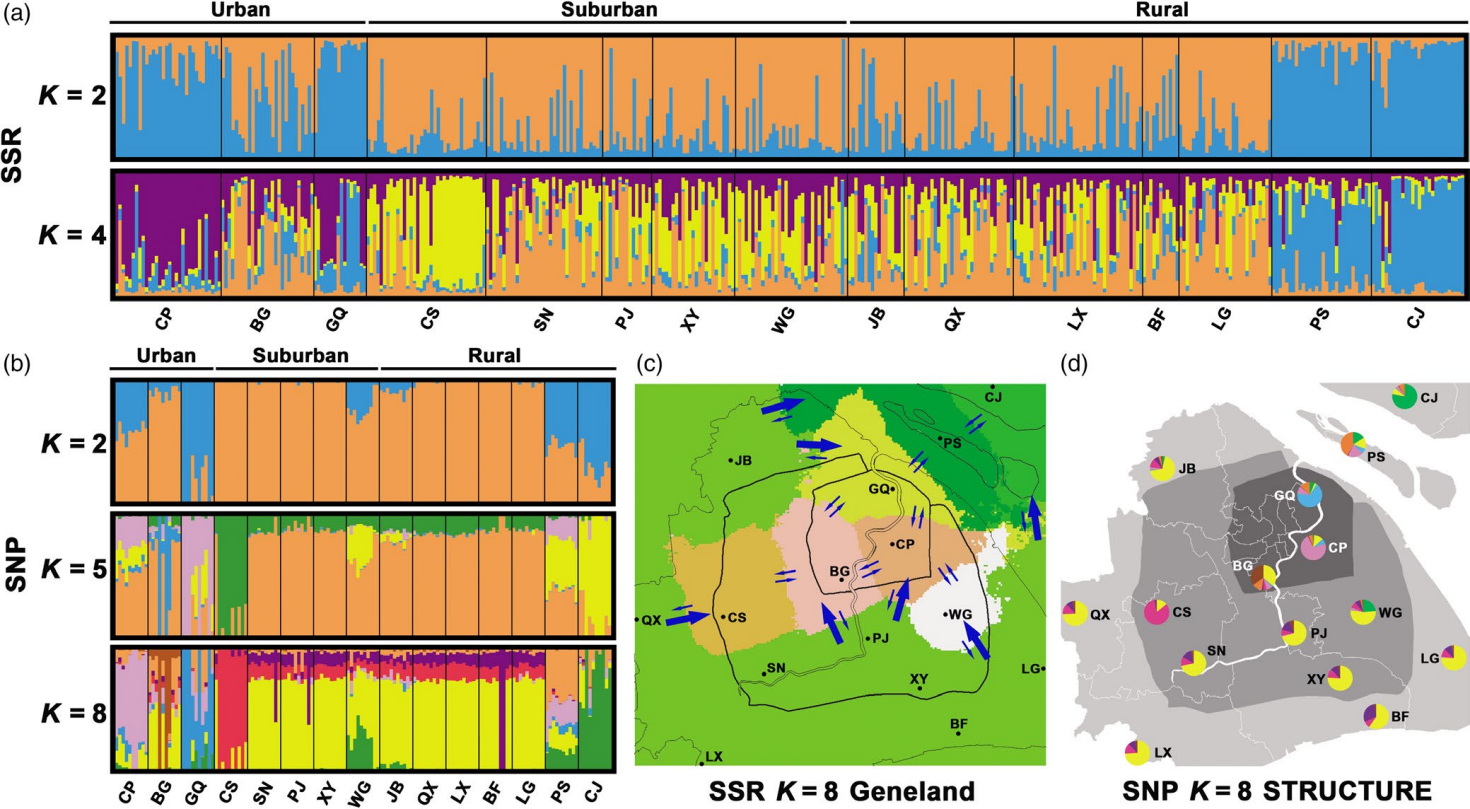
Population structure of *Pelophylax plancyi* analyzed using SSR and SNP data. Bar plots of *individual Q‐matrices* calculated by STRUCTURE using (a) the 13 SSR loci and (b) the 44,469 SNP loci with the local populations assigned in order of urban, suburban, and rural groups; (c) map of clusters simulated by 13 SSR loci in *Geneland* with asymmetrical gene flow illustration (bigger arrow—stronger gene flow; smaller arrow—weaker gene flow); (d) map of pie charts of *population Q‐matrix* simulated by 44,469 SNP loci in STRUCTURE. Site abbreviations correspond to those in Table 1

**Figure 5:**
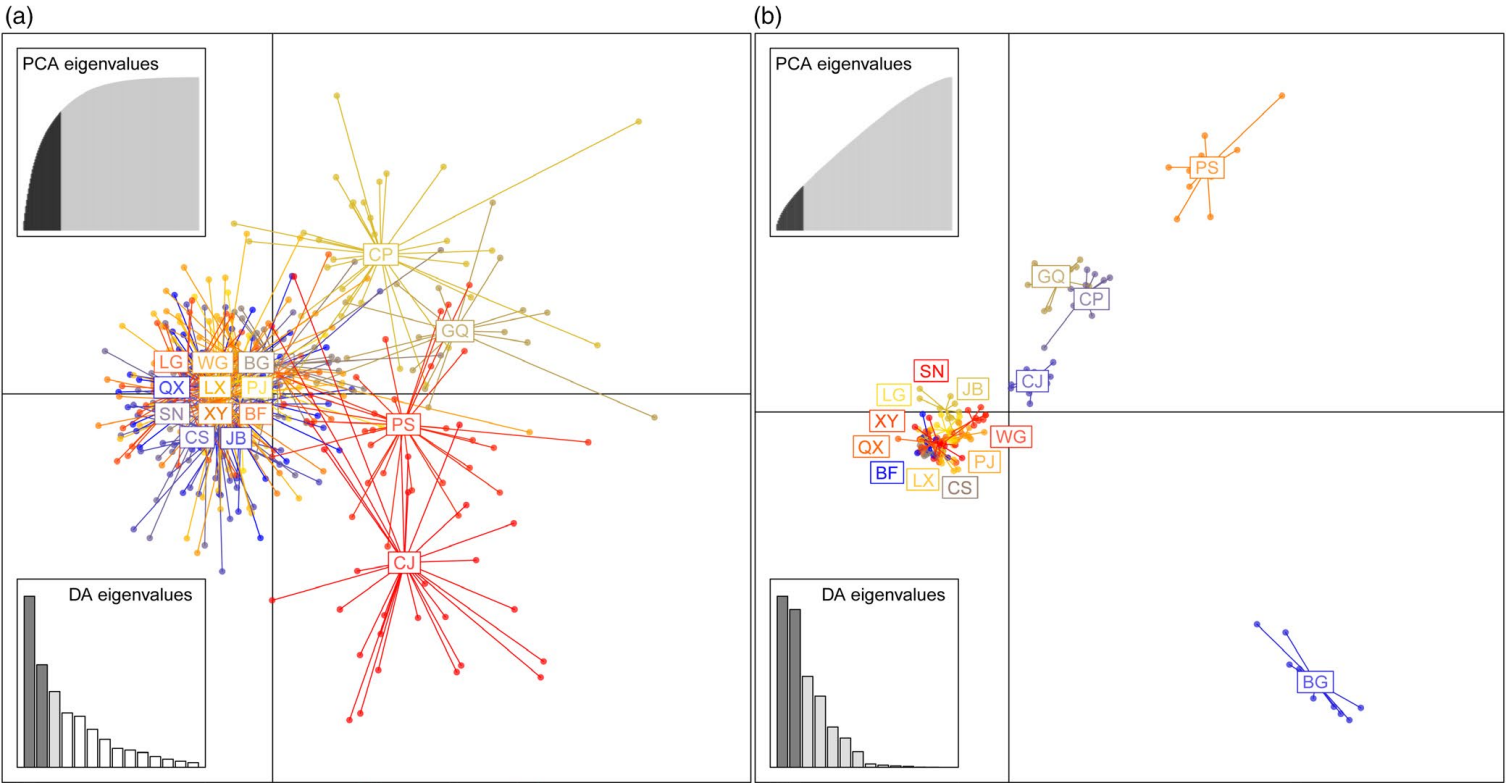
Scatterplots results from discriminant analysis of principal components (DAPC) for all 15 local *Pelophylax plancyi* populations using (a) the 13 SSR loci and (b) the 71,910 SNP loci. Insets represent the eigenvalues of retained principal components (top left), and the eigenvalues of discriminant functions (bottom left). Site abbreviations correspond to those in Table 1

STRUCTURE results for SSR data supported optimal numbers of clusters when *K* = 2 and *K* = 4 (Figure S5a). In both cases, the two island local populations (PS and CJ) and two urban local populations (GQ and CP) were grouped into differentiated clusters (Figure 4a). Furthermore, results from *Geneland* supported a more detailed eight‐cluster structure, including two island clusters (i.e., CJ and PS), three urban clusters (i.e., GQ, CP, and BG), two suburban clusters (i.e., CS and WG), and one big peripheral cluster containing all the other local populations (Figure 4c). Subsequent DAPC analysis retained 39 principal components (PCs; Figure S6), and the first two discriminant functions distinguished five clusters, including two urban local populations (CP and GQ), two island local populations (PS and CJ) and one big cluster of the remaining 11 local populations (Figure 5a).

In terms of the SNPs, STRUCTURE showed that the optimal *K* of genetic clusters was two, five, and eight (Figure S5b). When *K* was eight, local urban populations CP, BG, and GQ; the same two suburban populations CS and WG, and the two island populations PS and CJ were independent clusters; and all the other local populations were grouped into a single peripheral cluster (Figure 4b,d). In Admixture analysis, as the cross‐validation showed quite limited differences from *K* = 2 to *K* = 8 (Figure S7), the series of bar plots were displayed in Figure S8. Admixture gave the most consistent population structure with STRUCTURE results when *K* = 8 (Figure S8). In the DAPC analysis, 23 PCs were retained (Figure S9). Three urban local populations (GQ, CP, and BG) and the two island local populations (PS and CJ) were distinguished from the other local populations (Figure 5b), which was generally consistent with the other genetic model simulation results presented earlier.

Therefore, according to the analyses of both SSRs (*Geneland*) and SNPs (STRUCTURE), the 15 local populations were categorized into eight genetic clusters as follows: CP, BG, GQ, CS, WG, PS, CJ, and a peripheral cluster including the local populations of SN, PJ, XY, JB, QX, LX, BF, and LG (Figure 4).

### 3.6 Gene flow between genetic clusters

In terms of SSRs, all three runs converged in BayesAss (Figure S10). Regarding the SNP data, the three replicate runs showed consistent results, though did not converge as diagnosed by Tracer. The results of the SNP data indicated asymmetrical migrations from the peripheral cluster to most of the other genetic clusters (migration rates: 0.165–0.204), except for lower rates to GQ and CJ (Table S10). By contrast, the migration rates between the other seven genetic clusters or to the peripheral cluster were extremely low (0.004–0.052; Table S10). Results of the SSR data showed a similar pattern to that of the SNPs (Table S11).

### 3.7 Urbanization index and statistical analysis

According to the PCA of the seven landscape variables (Table S4), the UI values differed significantly among the 15 sampling sites (*t* = 21.816, *p* < .001). Linear regression model (LRM) showed that UI decreased significantly along the cityscape gradient from urban to rural areas (regression coefficient (RC) = −0.223, $R_{\text{adj}}^{2}$ = 0.321, *p* = .016; Table 1).

Moderate, but significant negative relationships between genetic variations and UI were detected at the local scale. *N*_H_ of mtDNA (RC = −4.986, $R_{\text{adj}}^{2}$ = 0.207, *p* = .050), *N*_A_ of SSRs (RC = −1.480, $R_{\text{adj}}^{2}$ = 0.259, *p* = .031), and *π* of SNPs (RC = −0.020, $R_{\text{adj}}^{2}$ = 0.320, *p* = .016) showed significant negative relationships with UI (Table 1). In terms of *N*_e_, although calculation by SSRs did not show a significant relationship with UI, SNP‐derived *N*_e_ was negatively related to UI at the local scale (RC = −1,812.0, $R_{\text{adj}}^{2}$ = 0.529, *p* = .002). Neither genetic diversity nor *N*_e_ were detected to have significant regression relationship with urbanization gradient at the cityscape scale.

## 4 DISCUSSION

Urbanization is one of the most important impacts of human development in the 21st century. Although populations have been shown to adapt to urban environments, urbanization frequently has negative effects on the movement, demography, and genetic diversity of populations (Miles et al., 2019). Therefore, understanding the evolutionary mechanisms by which urbanization and associated fragmentation affect populations can provide valuable knowledge about the conservation and management of native and non‐native populations alike (Lambert & Donihue, 2020). In this study, we presented the influence of the long‐term urbanizing process in Shanghai City on population genetics of *P. plancyi*. The ongoing population fragmentation of this anuran species with limited dispersal ability provides a good example to discuss the conservation biology of native wildlife in intensively urbanizing environments.

### 4.1 Demographic variation

Examining the demographic processes of species is essential for evaluating how urban development affects the historical and contemporary effective population sizes (*N*_e_), and consequently, causes different intensities of genetic drift between local populations in areas with different urbanization levels. In the lower Yangtze River areas, agricultural development and urbanization have had substantial environmental impacts since 2800–2200 B.P. (Atahan et al., 2008). During this period, agriculture underwent significant development in Shanghai (Wang et al., 1996; Figure 1). Both the SSR (Figure 3 and Table S5) and SNP (Table S7) data showed that bottleneck effects in all 15 local populations occurred during the agriculture‐dominated historical stage, several hundred years ago. Urbanization has been linked to agricultural intensification and the translocation of species (e.g., domesticated species and human commensals) since the Iron Age (Boivin et al., 2016). Both agriculture and translocated species can not only cause resource or habitat competition with local wildlife (McClure, 2013; Yeakel et al., 2014), but also degrade the suitability of the remaining habitat. In addition, a range of predator species, including humans, have constantly exploited amphibians for hundreds of years (Wells, 2007). Wildlife consumption is a tradition in many countries, and overexploitation remains one of the main threats to 78% of 437 vertebrate species (64% for 25 amphibian species studied) in China (Li & Wilcove, 2005). Therefore, similar to Harris et al. (2016), our data suggested that population decline of indigenous wildlife species might have been driven by historical agriculture‐dominated time periods when there was little urbanization, and not the recent fast urbanization typical of recent times.

Moreover, the SNP results further suggested that *N*_e_ values of all *P. plancyi* local populations are recovering from the bottleneck effects in recent years (Table S7). According to the recommended threshold of *N*_e_ ≥ 100 to avoid inbreeding depression (Frankham et al., 2014), all *P. plancyi* local populations still possess enough *N*_e_ to persist in the city. Lourenço et al. (2017) found that habitat patch size was the most significant variable positively associated with *N*_e_. In this study, all sampling sites were >10 ha and located in areas with relatively stable land‐use forms over the past few decades. Therefore, although modern urbanization might decrease gene flow between urban populations of wildlife species (Hamer & McDonnell, 2008; Lambert & Donihue, 2020), protecting the core habitat area (Cushman, 2006; Semlitsch, 2008) could be pivotal to maintain *N*_e_ and genetic diversity of urban populations at suitable levels for longer persistence.

### 4.2 Modern urbanization and genetic diversity of local populations

Higher genetic diversity is usually correlated with larger *N*_e_ and higher population fitness (Frankham, 2012; Reed & Frankham, 2003), and thus, is a fundamental indicator of population health and stability. Although genetic diversity of some species increased following urban facilitation models, genetic diversity of most studied animal species decreased in response to urbanization (e.g., the urban fragmentation model; Miles et al., 2019; Schmidt et al., 2020). These types of genetic diversity decline in response to urbanization were detected in all classes of vertebrates (Johnson & Munshi‐South, 2017). Dispersal ability is one of the most significant factors influencing the population genetics of wildlife in urban areas (Lambert & Donihue, 2020). Vertebrate species that walk tend to be more vulnerable to urbanization because of their obviously easier interrupted gene flow between populations (Medina et al., 2018; Schmidt et al., 2020).

In amphibians, their aquatic‐terrestrial biphasic life cycles and limited terrestrial dispersal ability usually cause them to be more vulnerable to the severe habitat fragmentation caused by modern urbanization (Semlitsch, 2008). Hitchings and Beebee (1997) found a positive correlation between genetic diversity and fitness of *Rana temporaria*, which were always the lowest in urbanized areas. In this study, the results from all three molecular markers indicated that comparable levels of *N*_e_ (Tables S5 and S7) and genetic diversity (Table 1) of *P. plancyi* were maintained in urban and suburban areas compared with rural areas. Meanwhile, although the UI within a 2‐km radius range of the core habitats showed moderate but significant impacts on the genetic diversity of *P. plancyi*, this impact was not significant along the urban‐to‐rural gradient (Table 1). These results implied that urbanization may have already influenced *P. plancyi* population fitness at the local scale but not yet at the cityscape scale. Therefore, providing better local habitats to maintain adequate *N*_e_ and genetic diversity in local populations will be essential to enable wildlife populations to persist in heavily urbanized areas (see "Urban amphibian species conservation" section for detailed discussion).

### 4.3 Environmental factors shaping the population genetic structure

Although the nonsignificant divergence of mtDNA between the 15 local populations supported a unified *P. plancyi* population (Figures S3 and S4) in Shanghai City, both SSR and SNP data showed a differentiated population structure (Figures 4 and 5, and S8). These results confirmed that both urbanization and geographic barriers can be significant factors in shaping the population structure of wildlife species.

Habitat fragmentation caused by urbanization can reduce gene flow, which can result in genetic isolation among local populations. The barrier to gene flow caused by impervious surfaces like buildings and roads in urban areas was confirmed in many vertebrate species, like the isolation effect of impervious surface in New York to white‐footed mouse (*Peromyscus leucopus*) populations (Munshi‐South et al., 2016), and the highway 101 to Wrentit (*Chamaea fasciata*) and side‐blotched lizard (*Uta stansburiana*) populations in Los Angeles (Thomassen et al., 2018). Regarding amphibians, fragmentation effects caused by urban infrastructures could be even more significant. For example, genetic structures of *L. sylvaticus* populations can be quite homogenous when natural or artificial habitats are available for adults and juveniles to move between sampling sites in urban areas (Furman et al., 2016; Homola et al., 2019), but the existence of impervious surfaces like buildings and roads can significantly decrease gene flow and cause genetic subdivision among fragmented populations (Crosby et al., 2009; Richardson, 2012). Similar results were also reported in *A. tigrinum* populations in New York City (McCartney‐Melstad et al., 2018). As for *P. plancyi* in this study, among the eight recognized genetic clusters, six occurred in the mainland area of the city, and the spatial distribution pattern was consistent with the urbanization gradient (Figure 4c,d). Connected agricultural lands across the vast rural areas remain the optimal habitat for amphibians in Shanghai City (Li et al., 2019), supported by the existence of one big peripheral cluster and the asymmetrical gene flow mainly from the peripheral cluster to other mainland clusters (Figure 4c). Zhang et al. (2016) reported a positive influence of agricultural and forest land coverage on the *P. plancyi* population size at the local scale (i.e., a 2‐km radius). Yue (2019) found that *P. plancyi* was sensitive to the existence of paved roads in urban areas. More urbanized areas are usually accompanied by less agricultural and forest land coverage, and denser paved roads, which supported the distribution pattern of the six genetic clusters in mainland Shanghai City (Figure 4).

Despite clear historical impacts on *P. plancyi* populations, the correlation between the distribution of the mainland genetic clusters with modern city development (Figure 4c,d) suggests that 40 years of rapid modern urbanization has already had a fast and significant impact on the genetic structure of the *P. plancyi* population in Shanghai City. Compared with the *S. salamandra* data from Oviedo (mean SSR pairwise *F*_ST_: 0.106 ± 0.039; Lourenço et al., 2017) or the *Plethodon cinereus* data from Montreal (0.064 ± 0.038; Noël et al., 2007), the degree of differentiation among *P. plancyi* local populations was still moderate (Table S9). However, given the relatively short period of rapid modern urbanization in Shanghai City, the intensity of differentiation was considerable, and even stronger than that caused by the island isolation effect (Table S9). A similar situation was reported in *D. fuscus* in New York City (Munshi‐South et al., 2013). Therefore, both time since isolation and the intensity of urbanization can determine the speed and degree of genetic differentiation.

Geographical barriers like islands and big rivers are common and important factor causing isolation of wildlife populations. The isolation effect caused by islands was obvious, as revealed by two island clusters (i.e., CJ and PS). However, we did not observe significant genetic differentiation between the west and east sides of the Huangpu River (Figures 4 and 5). Big rivers are often regarded as barriers to amphibian species with difficulties to traverse large waterways (Li et al., 2009; Moraes et al., 2016). However, in some studies, rivers appeared to facilitate gene flow within amphibian species (Mikulíček & Pišút, 2012; Spear et al., 2005). For example, Mikulíček and Pišút (2012) suggested that the effect of rivers on genetic differentiation of amphibian populations is not uniform, but depends on the characteristics of the river and specific traits of the wildlife species studied. Meanwhile, effects of natural barriers are usually influenced by human activities in an urbanizing environment. The Huangpu River is the biggest river and the main shipping channel in Shanghai City. Most of the riverbank has been lined by a solid up‐right concrete wall since the 1990s, which is not suitable for *P. plancyi* to pass through. However, given that the Huangpu River is not long, corridors at headwaters might still exist. Moreover, the ~30 years of riverbank building might not be sufficient to cause divergence of the local populations on both sides. Therefore, the effect of the Huangpu River on the population genetics of this species in Shanghai City requires further population monitoring and landscape genetic studies. Joint effects between natural barriers and urbanization to the population fragmentation of urban wildlife species should be considered.

### 4.4 Urban amphibian species conservation

Protecting wildlife in urbanized areas is important not only for conservation, but also for a better and healthier urban ecosystem (Yang et al., 2015). Amphibians are good indicators of habitat alterations and thus have high conservation values in urban environments. Natural or artificial ponds and wetlands with clean water, a high coverage of floating leaved vegetation, and traversable aquatic–terrestrial interfaces are typical suitable microhabitats for *P. plancyi* (Huang et al., 2018; Yue, 2019). Such kinds of suitable pond and wetland microhabitats are widely spread across the city (Li et al., 2017), resulting in *P. plancyi* being the most abundant and widely distributed anuran in Shanghai (Gu, 2015; Huang et al., 2018; Yue, 2019). In this study, although genetic isolation caused by urbanization was confirmed, the recovering *N*_e_ values of the 15 sampled local populations (Table S7) suggest the possibility of population conservation for long‐term persistence in heavily urbanized environments. However, as local populations in urban, suburban, and rural areas exhibited differentiated genetic structure (Figure 4), strategies for amphibian species conservation need to be specific to the different areas of the city.

Many studies have suggested the importance of protecting the core habitat area based on philopatry and the limited migration ability of adult amphibians (reviewed by Cushman, 2006), especially for anuran pond breeders with complex aquatic–terrestrial habitat use patterns (Semlitsch, 2008). Indeed, the five genetic clusters located in urban and suburban areas (Figure 4c) are typical isolated local populations located in highly fragmented urban habitat remnants where the success of juvenile dispersal can be low. Protecting the core habitat areas would help preserve the source for these populations, which would also be beneficial to the entire urban wildlife community. Although no direct data of the migration distance of *P. plancyi* adults are available, Smith and Green (2005) reported that >60% (33/53) of anuran species worldwide moved < 500 m. Drinnan (2005) suggested that the discrete habitat remnant size should be >4 ha for bird and frog species conservation in Australia. Therefore, protecting core habitats for amphibians might not require large territories in heavily urbanized areas and could reflect a good balance between municipal economy/livelihood land‐use planning and wildlife conservation. Urban green spaces, including parks and public woods, are highly valued refugees for birds (Yang et al., 2015; Ye, 2016) and amphibians (Li et al., 2018; Zhang et al., 2016). There are more than 217 parks in Shanghai City, and the average area of each park is 12.23 (± 29.28) ha, with a median value of 3.8 ha (Shanghai Landscaping & City Appearance Administrative Bureau, unpublished data). In this study, 10 sampling sites were located in parks and gardens (Table 1) with an area each > 10 ha. The recovering *N*_e_ (Table S7) and comparable genetic diversity (Table 1) of the local populations in these sampling sites compared with the other five local populations suggest that well‐designed artificial habitats in urban green spaces would be suitable core habitats for wildlife species with limited dispersal ability.

Although core habitat area protection might be effective for urban amphibian conservation, a larger scale habitat matrix to support larger populations with rich genetic exchange will be more precious (Semlitsch, 2008). A notable result of this study was the existence of a big peripheral genetic cluster located mainly in the rural areas of mainland Shanghai City, which included eight local populations (Figure 4). Agricultural lands in rural areas are traditionally the most optimal habitats for wildlife conservation (SFB, 2004). Lower urbanization levels and larger connected habitat areas compared with urban and suburban habitat patches are the main advantages of rural agricultural lands in terms of supporting larger wildlife populations and higher species diversity (Li et al., 2019). However, Liu et al. (2020) reported that ~70% of urban growth since 1990s occurred at the expense of agricultural lands. Therefore, although urbanization will continue to intensify (Kuang et al., 2014), as a part of the city development plan facing 2035, rural agricultural lands of Shanghai are under protection, which provides a good foundation to protect source habitats for the biodiversity conservation in the city.

Maintaining gene flow between populations is important for the long‐term persistence of populations (Miles et al., 2019). In this study, the asymmetrical gene flow from the peripheral cluster to urban and suburban clusters (Table S10) suggested that corridors between rural and urban local populations exist but are more restricted between urban local populations. For *P. plancyi* and other amphibian species in Shanghai City, the importance of environmental factors, such as forest and wetland composition (Zhang et al., 2016) and configuration structures (Li et al., 2018) to promote dispersal and migration between breeding sites, has been reported, and all improve gene flow among discrete local populations in urban areas. In fact, an ecological corridor network project (SMPG, 2018) has been planned and has started to encompass the entire city territory to improve wildlife species distribution and conservation in this area. However, how to keep or establish corridors to support gene flow among local populations in both rural source habitats and urban habitat remnants can be specific to a particular species in a specific environment (Jarvis et al., 2019), especially for terrestrial locomotive species (Medina et al., 2018). Therefore, to develop a well‐designed urban wildlife species conservation plan, fundamental ecological information of the species is essential.

To the majority of amphibian species including *P. plancyi*, knowledge of their dispersal behavior is limited (Smith & Green, 2005). In addition, given the possibility of delayed biological responses to habitat alteration (e.g., Keyghobadi et al., 2005) and the ongoing rapid urbanization of developing countries, long‐term monitoring of population genetics is necessary for conservation management. The fast development of next‐generation sequencing provides powerful tools to detect functional genomic adaptations of wildlife to urbanization (e.g., Harris & Munshi‐South, 2017) and to apply landscape genetics analyses to determine how landscape influences this evolutionary process (e.g., McCartney‐Melstad et al., 2018; Munshi‐South et al., 2016). Such information enables us to further understand the mechanisms underlying the effects of urbanization on the evolution of wildlife populations.

## 5 CONCLUSIONS AND IMPLICATIONS

In this study, we confirmed that long‐term urbanization could influence the population genetics of native wildlife species in different stages during a process of thousands of years, resulting in profound evolutionary and conservational consequences to the urban wildlife. Population bottlenecks of wildlife species with limited dispersal ability like *P. plancyi* could have already begun more than one thousand years ago during the historical low‐level urbanization dominated by agriculture, while the intensive modern urbanization for dozens of years more significantly influenced the gene flow among local populations and caused urban wildlife population differentiation along the urban‐to‐rural gradient. However, urban local populations can still keep comparable *N*_e_ and genetic diversity with those in rural areas, which suggested that protecting core habitat areas would be an effective method to maintain local populations of urban wildlife species with limited dispersal abilities. Nevertheless, for long‐term conservation planning and management plan, a corridor network to maintain gene flow between local populations in the large rural agricultural areas and discrete urban local populations is imperative.

## CONFLICT OF INTEREST

None declared.

## Supporting information

Supplementary Material

## Data Availability

Data of *Cyt‐b* haplotypes, SSR genotypes, and SNP loci used in this study are accessible on Figshare (https://doi.org/10.6084/m9.figshare.11848374), and ddRAD reads in the study are available on NCBI’s Short‐read Archive (SRA) with accession number PRJNA606868, to be published after a decision has been made on the manuscript.
