# Supplementary material for: Long‐term urbanization impacts the eastern golden frog (Pelophylax plancyi) in Shanghai City: Demographic history, genetic structure, and implications for amphibian conservation in intensively urbanizing environments
Source: Evol Appl. 2020 Nov 7;14(1):117–35. doi: 10.1111/eva.13156 (PMC7819575; doi:10.1111/eva.13156)
Supplement: Supplementary file 1 — Supplementary Material [file EVA-14-117-s001.docx]

**Supporting information**

**Article title:** Long-term urbanization impacts the eastern golden frog (*Pelophylax plancyi*) in Shanghai City: demographic history, genetic structure, and implications for amphibian conservation in intensively urbanizing environments

**Appendix S1** SSR loci extraction from RADseq data

**Table S1** Characteristics of landscape and *P. plancyi* sampling sizes (*N*) at the 15 sampling sites

**Table S2** Details of the primers of the 15 SSR loci used in this study

**Table S3** Input parameters in the R package *VarEff* for each local population

**Table S4** Principal component analysis scores of the seven principal components

**Table S5** Gelman-Rubin test and variables calculated by *VarEff* using the 13 SSR loci for the 15 local populations of *P. plancyi*

**Table S6** Logistic regression posterior probabilities given for the three tested demographic scenarios in each local population by DIYABC

**Table S7** Posterior distribution of parameters (median value) for demographic variation in *P. plancyi* local populations estimated under the optimal scenario of SNP data in DIYABC

**Table S8** Bias and error estimates of posterior parameter distributions for the optimal scenario in each local population

**Table S9** Pairwise *F*_ST_ calculated by the 71,910 SNP loci (below diagonal) and the 13 SSR loci (above diagonal) between local populations of *P. plancyi*

**Table S10** Recent migration rates between each pair of genetic clusters of *P. plancyi* estimated from the 1,611 SNP loci using BayesAss3

**Table S11** Recent migration rates between each pair of genetic clusters of *P. plancyi* estimated from the 13 SSR loci using BayesAss3

**Figure S1** Model checking plots of PCA scores for the 15 local populations

**Figure S2** Network of 68 *Cyt-b* gene haplotypes of *P.* *plancyi*

**Figure S3** Maximum likelihood tree constructed based on the *Cyt-b* gene data

**Figure S4** Bayesian tree constructed based on the *Cyt-b* gene data

**Figure S5** Estimations of *ΔK* for (a) SSRs and (b) SNPs in STRUCTURE analysis

**Figure S6** Number of principal components retained for the DAPC analysis of SSRs

**Figure S7** Cross-validation errors from Admixture for *K* from 1 to 15

**Figure S8** Bar plots of population structure analyses using Admixture

**Figure S9** Number of principal components retained for the DAPC analysis of SNPs

**Figure S10** Plot of the MCMC chain convergence calculated in BayesAss by SSR data

**Appendix S1** SSR loci extraction from RADseq data

The high-quality RADseq data of the sample ‘LX01’ was used for the filtering of SSR loci. We used FLASH v1.2.11 (Magoc & Salzberg, 2011) to merge paired-end reads from RADseq data with default parameters. The reads that could not be merged were discarded, and only the merged sequences > 200 bp in length were retained for further analysis. CD-HIT v4.7 (Fu, Niu, Zhu, Wu, & Li, 2012) was used to remove the sequence redundancy of merged reads with parameters for sequence identity threshold (*c* = 1), alignment coverage for the longer sequence (*aL* = 1), and alignment coverage for the shorter sequence (*aS* = 1). Using MISA (Thiel, Michalek, Varshney, & Graner, 2003), we selected candidate SSR loci. We defined that the putative loci sequences would be retained if: (1) the sequence contained more than six repeats of a dinucleotide motif, or more than five repeats of a trinucleotide motif; (2) there should be at least 100 bp between two putative loci. Considering the expense of running the experiments, we randomly selected 15 candidate loci for primer synthesis using Primer Premier v5.0 (Premier Biosoft International, Palo Alto, CA, USA), and experimental verification was performed following the description in Materials and Methods in the main text. We eventually obtained another four polymorphic loci that had specific amplification with good PCR quality. The detailed information of the primers is listed in Table S2.

**Table S1** Characteristics of landscape and *P. plancyi* sampling sizes (*N*) at the 15 sampling sites

| Sampling site | Type | *N* | PIS | NP | ED | LSI | AREA | SHAPE | ENN | UI |
| --- | --- | --- | --- | --- | --- | --- | --- | --- | --- | --- |
| Century Park (CP) | Urban | 32 | 0.748 | 478 | 235.650 | 20.493 | 2.260 | 1.782 | 43.302 | 0.577 |
| Shanghai Botanical Garden (BG) | Urban | 28 | 0.787 | 351 | 165.435 | 14.723 | 3.079 | 1.561 | 66.513 | 0.800 |
| Gongqing Forest Park (GQ) | Urban | 16 | 0.796 | 212 | 107.598 | 9.950 | 5.088 | 1.571 | 65.070 | 1.000 |
| Chenshan Botanical Garden (CS) | Suburban | 36 | 0.356 | 402 | 173.882 | 15.415 | 2.691 | 1.613 | 59.011 | 0.669 |
| Songnan Country Park (SN) | Suburban | 35 | 0.181 | 914 | 212.095 | 18.569 | 1.185 | 1.386 | 44.633 | 0.299 |
| Pujiang Country Park (PJ) | Suburban | 15 | 0.456 | 1478 | 306.515 | 26.330 | 0.732 | 1.422 | 42.585 | 0.109 |
| Xiangyang Village (XY) | Suburban | 25 | 0.194 | 521 | 232.532 | 20.231 | 2.079 | 1.565 | 42.840 | 0.406 |
| Wuzao Gang (WG) | Suburban | 34 | 0.284 | 763 | 302.008 | 25.893 | 1.412 | 1.490 | 38.172 | 0.202 |
| Jiabei Country Park (JB) | Rural | 17 | 0.183 | 1,360 | 329.850 | 28.169 | 0.793 | 1.568 | 38.766 | 0.072 |
| Qingxi Country Park (QX) | Rural | 33 | 0.178 | 1,332 | 305.254 | 26.207 | 0.812 | 1.458 | 40.530 | 0.087 |
| Langxia Country Park (LX) | Rural | 39 | 0.185 | 1,680 | 307.570 | 26.404 | 0.643 | 1.370 | 39.085 | 0.000 |
| Bay Forest Park (BF) | Rural | 11 | 0.186 | 411 | 148.900 | 13.361 | 2.637 | 1.546 | 52.970 | 0.623 |
| Lingang Town (LG) | Rural | 28 | 0.174 | 441 | 172.409 | 15.292 | 2.454 | 1.496 | 48.195 | 0.530 |
| Panshi Town, Changxing Island (PS) | Rural | 30 | 0.127 | 819 | 196.859 | 17.269 | 1.316 | 1.434 | 39.442 | 0.319 |
| Chenjia Town, Chongming Island (CJ) | Rural | 28 | 0.273 | 681 | 212.324 | 18.537 | 1.580 | 1.514 | 39.922 | 0.376 |

Type - the relative location of the local populations in the city; *N* - sampling size; PIS - percentage of impervious surface; NP - number of patches; ED - edge density; LSI - landscape shape index; AREA - mean patch area; SHAPE - shape index; ENN - Euclidean nearest-neighbor distance; UI - urbanization index.

**Table S2** Details of the primers of the 15 SSR loci used in this study

| Locus | Repeat Motif | Forward Primer (5'-3') | Reverse Primer (5'-3') | Annealing T (°C) | Reference |
| --- | --- | --- | --- | --- | --- |
| Pn103 | GT | TAGGCAGATTTGTGGCATTT | AGAGGGATTCTCCTGTTATG | 55 | (Du et al., 2012) |
| Pn105 | AC | ATACAGACCTTGGTAAGTTTT | GTTTATTCTATCAGTTAGTGGAG | 55 | (Du et al., 2012) |
| Pn204 | GA | ATCCGCCGCTTATGACGAGG | CAGCCTGCTTAGAAGAAGAAA | 55 | (Du et al., 2012) |
| Pn214 | AG | AGAATGAAAAGAATAGTAGGGA | AGATTGTAAGAGGGAACAGTAGTCA | 55 | (Du et al., 2012) |
| Pn221 | GA | AGGCAGAACATAAAGACGCA | GCCAGCACATTGGGGTAGAG | 60 | (Du et al., 2012) |
| Pla08 | AC | ACTAAACACTCCTCTGCGACCTG | CGGTTACTTTACCTTGTAGAATTGC | 60 | (Dai & Zhou, 2009) |
| Pla13 | GA | TGCTCTTATCACCACAAACTCTCAT | CAGTCTCACCATCAGAACTCTAGAT | 52 | (Dai & Zhou, 2009) |
| Pla34 | CT | TCTATGTTTAGCTCCAATGCTGG | GCTCATGGCATCATACTCCTTTAGG | 55 | (Dai & Zhou, 2009) |
| Pla36 | TC | CTTGTGCTATGAGTTCTCGCACTC | GTCTTGAAACTTTGCACAGTCTATG | 60 | (Dai & Zhou, 2009) |
| Pla70 | TG | ATCCTGGTTCAAAGACACC | ACGCACCTATTTCCATCAC | 60 | (Dai & Zhou, 2009) |
| Pla96 | GA | GCAATGCCTTGGTAGTTCA | AAGGAGCGACCATCAATCT | 55 | (Dai & Zhou, 2009) |
| W02 | CA | CTTTACGCACAATCCTGA | AACGACCGCTCTGAAACC | 55 | This study |
| W06 | CT | ACCTGTGCCGAGATGGAC | GTAGGCTATTTGAATGGG | 55 | This study |
| P05 | GAT | TGCCCCTTTAATGCAGAACA | GCACCTCATCCCTGGAGAAT | 55 | This study |
| H10 | CT | AGTAGCCACCACCAGCACCA | GGAGACAAGCAATTAATAATTGATG | 55 | This study |

**Table S3** Input parameters in the R package *VarEff* for each local population

| Local population | NBLOC | JMAX | DMAX | NBAR | VARP1 | RHOCORN | GBAR | VARP2 | Diagonale | AccRate |
| --- | --- | --- | --- | --- | --- | --- | --- | --- | --- | --- |
| CP | 13 | 3 | 9 | 2,370 | 3 | 0.5 | 5,000 | 3 | 0.5 | 0.25 |
| BG | 13 | 3 | 14 | 2,419 | 3 | 0.5 | 5,000 | 3 | 0.5 | 0.25 |
| GQ | 13 | 3 | 10 | 2,499 | 3 | 0.5 | 5,000 | 3 | 0.5 | 0.25 |
| CS | 13 | 3 | 12 | 2,022 | 3 | 0.5 | 5,000 | 3 | 0.5 | 0.25 |
| SN | 13 | 3 | 12 | 2,341 | 3 | 0.5 | 5,000 | 3 | 0.5 | 0.25 |
| PJ | 13 | 3 | 13 | 2,297 | 3 | 0.5 | 5,000 | 3 | 0.5 | 0.25 |
| XY | 13 | 3 | 14 | 2,015 | 3 | 0.5 | 5,000 | 3 | 0.5 | 0.25 |
| WG | 13 | 3 | 14 | 2,319 | 3 | 0.5 | 5,000 | 3 | 0.5 | 0.25 |
| JB | 13 | 3 | 12 | 2,525 | 3 | 0.5 | 5,000 | 3 | 0.5 | 0.25 |
| QX | 13 | 3 | 10 | 2,108 | 3 | 0.5 | 5,000 | 3 | 0.5 | 0.25 |
| LX | 13 | 3 | 10 | 2,075 | 3 | 0.5 | 5,000 | 3 | 0.5 | 0.25 |
| BF | 13 | 3 | 13 | 2,384 | 3 | 0.5 | 5,000 | 3 | 0.5 | 0.25 |
| LG | 13 | 3 | 13 | 2,473 | 3 | 0.5 | 5,000 | 3 | 0.5 | 0.25 |
| PS | 13 | 3 | 13 | 2,931 | 3 | 0.5 | 5,000 | 3 | 0.5 | 0.25 |
| CJ | 13 | 3 | 13 | 3,468 | 3 | 0.5 | 5,000 | 3 | 0.5 | 0.25 |

NBLOC - the number of SSR loci; JMAX - the number of times that the effective population size changed in the past; DMAX - maximum distance (in motifs) between alleles; NBAR - the prior for effective population size; VARP1 - prior variances for effective population size; RHOCORN - correlation between effective population size among successive intervals; GBAR - the number of generations since population origin; VARP2 - prior variances for number of generations; Diagonale - a smoothing parameter; AccRate - acceptance rate. Site abbreviations correspond to those in Table 1 in the main text.

**Table S4** Principal component analysis scores of the seven principal components

|  | PC1 | PC2 | PC3 | PC4 | PC5 | PC6 | PC7 |
| --- | --- | --- | --- | --- | --- | --- | --- |
| PIS | 0.295 | 0.562 | -0.523 | 0.538 | 0.155 | 0.103 | -0.001 |
| NP | -0.410 | 0.053 | -0.429 | -0.172 | 0.237 | -0.748 | -0.001 |
| ED | -0.411 | 0.360 | -0.080 | -0.179 | -0.332 | 0.233 | -0.706 |
| LSI | -0.411 | 0.360 | -0.082 | -0.179 | -0.327 | 0.234 | 0.708 |
| AREA | 0.432 | 0.006 | -0.098 | -0.030 | -0.790 | -0.423 | 0.003 |
| SHAPE | 0.255 | 0.648 | 0.547 | -0.301 | 0.230 | -0.266 | 0.000 |
| ENN | 0.395 | -0.043 | -0.469 | -0.725 | 0.159 | 0.267 | -0.002 |
| Standard Deviation | 2.224 | 1.051 | 0.795 | 0.390 | 0.305 | 0.271 | 0.002 |
| Eigenvalue | 4.945 | 1.105 | 0.632 | 0.152 | 0.093 | 0.073 | 0.000 |
| Proportion of Variance | 0.706 | 0.158 | 0.090 | 0.022 | 0.013 | 0.010 | 0.000 |
| Cumulative Proportion | 0.706 | 0.864 | 0.954 | 0.976 | 0.990 | 1.000 | 1.000 |

PIS - percentage of impervious surface; NP - number of patches; ED - edge density; LSI - landscape shape index; AREA - mean patch area; SHAPE - shape index; ENN - euclidean nearest-neighbor distance.

**Table S5** Gelman-Rubin test and variables calculated by *VarEff* using the 13 SSR loci for the 15 local populations of *P. plancyi*

| Local population | PSRF | *N*_max_ | *T*_bot_ | *MG* | *T*_MG_ | *N*_e_ |
| --- | --- | --- | --- | --- | --- | --- |
| CP | 1.01 | 2,158.82 (1,009.28 - 4,975.55) | 1,536 | 5.64 | 335 | 428.42 (162.18 - 749.53) |
| BG | 1.01 | 2,067.51 (1,009.15 - 4,917.34) | 1,669 | 10.42 | 198 | 218.64 (92.95 - 403.40) |
| GQ | 1.00 | 2,367.08 (1,378.09 - 5,292.12) | 588 | 13.75 | 239 | 274.24 (112.17 - 497.22) |
| CS | 1.00 | 2,150.38 (794.37 - 9,403.88) | 4,123 | 2.68 | 358 | 373.15 (121.13 - 570.32) |
| SN | 1.07 | 3,347.92 (1,767.60 - 6,732.85) | 1,828 | 7.96 | 378 | 345.16 (145.25 - 584.85) |
| PJ | 1.02 | 3,545.69 (1,553.89 - 8,743.56) | 2,871 | 4.72 | 1,065 | 563.66 (258.11 - 817.05) |
| XY | 1.00 | 2,698.88 (804.49 - 7,891.01) | 3,003 | 4.28 | 907 | 372.49 (201.74 - 639.13) |
| WG | 1.00 | 5,212.19 (2,574.87 - 10,401.61) | 2,901 | 7.57 | 590 | 329.21 (142.23 - 544.86) |
| JB | 1.01 | 3,502.49 (1,989.73 - 6,576.56) | 1,894 | 10.56 | 315 | 334.01 (137.07 - 589.18) |
| QX | 1.12 | 2,626.91 (1,329.34 - 5,478.17) | 1,664 | 9.34 | 259 | 281.49 (111.67 - 494.80) |
| LX | 1.07 | 2,163.67 (991.23 - 4,887.47) | 1,512 | 7.81 | 217 | 340.07 (126.66 - 616.87) |
| BF | 1.05 | 2,674.37 (1,426.69 - 5,156.11) | 1,558 | 7.70 | 282 | 310.80 (132.06 - 557.56) |
| LG | 1.00 | 3,642.85 (2,130.40 - 7,261.65) | 2,324 | 7.56 | 458 | 348.03 (145.95 - 612.35) |
| PS | 1.04 | 3,947.63 (2,459.37 - 7,665.43) | 1,728 | 8.46 | 728 | 569.41 (233.83 - 966.78) |
| CJ | 1.02 | 4,065.67 (2,471.13 - 7,763.79) | 1,516 | 8.71 | 452 | 444.72 (170.46 - 787.33) |

PSRF - potential scale reduction factor in Gelman-Rubin test; *N*_max_ - the ancestral maximum effective population size; *T*_bot_ - time point when bottleneck began; *MG* - maximum magnitude of bottleneck effect (the number of individuals decreasing per generation/year); *T*_MG_ - time point when bottleneck showed strongest effect on the local population; *N*_e_ - contemporary effective population size. The values in parentheses represented the 5% and 95% quantiles of each parameter. Site abbreviations correspond to those in Table 1 in the main text.

**Table S6** Logistic regression posterior probabilities given for the three tested demographic scenarios in each local population by DIYABC

| Local Population | Scenario 1 | Scenario 2 | Scenario 3 | Error rate |
| --- | --- | --- | --- | --- |
| CP | 0.0274 (0.0244, 0.0304) | 0.9726* (0.9696, 0.9756) | 0.0000 (0.0000, 0.0000) | 0.040 |
| BG | 0.0127 (0.0104, 0.0149) | 0.9873* (0.9851, 0.9896) | 0.0000 (0.0000, 0.0000) | 0.041 |
| GQ | 0.0094 (0.0065, 0.0124) | 0.9905* (0.9876, 0.9935) | 0.0000 (0.0000, 0.0001) | 0.044 |
| CS | 0.0452 (0.0000, 0.2170) | 0.7263* (0.6613, 0.7914) | 0.2285 (0.0529, 0.4041) | 0.243 |
| SN | 0.1881 (0.1830, 0.1932) | 0.8119* (0.8068, 0.8170) | 0.0000 (0.0000, 0.0000) | 0.216 |
| PJ | 0.0762 (0.0723, 0.0800) | 0.9238* (0.9200, 0.9277) | 0.0000 (0.0000, 0.0000) | 0.079 |
| XY | 0.1155 (0.1112, 0.1198) | 0.8845* (0.8802, 0.8888) | 0.0000 (0.0000, 0.0000) | 0.167 |
| WG | 0.1931 (0.1878, 0.1984) | 0.8069* (0.8016, 0.8122) | 0.0000 (0.0000, 0.0000) | 0.106 |
| JB | 0.1795 (0.1744, 0.1845) | 0.8205* (0.8155, 0.8256) | 0.0000 (0.0000, 0.0000) | 0.185 |
| QX | 0.1547 (0.1497, 0.1596) | 0.8453* (0.8404, 0.8503) | 0.0000 (0.0000, 0.0000) | 0.133 |
| LX | 0.2583 (0.2527, 0.2639) | 0.7417* (0.7361, 0.7473) | 0.0000 (0.0000, 0.0000) | 0.286 |
| BF | 0.0548 (0.0513, 0.0583) | 0.9452* (0.9417, 0.9487) | 0.0000 (0.0000, 0.0000) | 0.039 |
| LG | 0.0771 (0.0731, 0.0811) | 0.9229* (0.9189, 0.9269) | 0.0000 (0.0000, 0.0000) | 0.071 |
| PS | 1.0000* (1.0000, 1.0000) | 0.0000 (0.0000, 0.0000) | 0.0000 (0.0000, 0.0000) | 0.000 |
| CJ | 0.0071 (0.0053, 0.0090) | 0.9887* (0.9842, 0.9932) | 0.0041 (0.0001, 0.0082) | 0.041 |

Scenario 1 - population expansion; Scenario 2 - population recovery; Scenario 3 - population contraction; Error rate - posterior predictive error rate given for the optimal scenario in each local population. * - the optimal scenario for each local population. The values in parentheses represent 95% confidence intervals. Site abbreviations correspond to those in Table 1 in the main text.

**Table S7** Posterior distribution of parameters (median value) for demographic variation in *P. plancyi* local populations estimated under the optimal scenario of SNP data in DIYABC

| Local population | Scenario | *N*_anc_ | *T*_anc_ | *N*_bot_ | *T*_bot_ | *N*_e_ |
| --- | --- | --- | --- | --- | --- | --- |
| CP | 2 | 4,880 (1,090 - 9,430) | 2,730 (824 - 4,750) | 54.1 (11.9 - 146) | 388 (174 - 491) | 376 (153 - 652) |
| BG | 2 | 5,130 (1,110 - 9,490) | 2,810 (816 - 4,760) | 45.6 (7.53 - 191) | 400 (187 - 493) | 231 (88.4 - 465) |
| GQ | 2 | 4,430 (821 - 9,390) | 3,040 (962 - 4,810) | 49.2 (5.98 - 283) | 410 (196 - 493) | 188 (62.2 - 411) |
| CS | 2 | 8,320 (3,700 - 9,890) | 101 (15 - 728) | 360 (43.5 - 901) | 73.4 (2.06 - 390) | 2,010 (274 - 7,620) |
| SN | 2 | 5,690 (2,120 - 9,570) | 3,520 (1,320 - 4,850) | 457 (177 - 715) | 357 (142 - 488) | 1,750 (724 - 2,800) |
| PJ | 2 | 5,350 (1,430 - 9,520) | 3,220 (1,120 - 4,820) | 244 (71.4 - 465) | 378 (164 - 490) | 876 (362 - 1,400) |
| XY | 2 | 5,440 (1,620 - 9,510) | 3,390 (1,240 - 4,840) | 369 (121 - 631) | 371 (150 - 489) | 1,170 (465 - 1,920) |
| WG | 2 | 5,860 (2,190 - 9,570) | 3,310 (1,190 - 4,820) | 293 (119 - 448) | 371 (155 - 488) | 1,930 (831 - 3,120) |
| JB | 2 | 5,650 (2,070 - 9,520) | 3,460 (1,360 - 4,850) | 420 (170 - 651) | 364 (147 - 487) | 1,740 (719 - 2,790) |
| QX | 2 | 5,570 (1,840 - 9,560) | 3,530 (1,360 - 4,860) | 385 (150 - 600) | 361 (140 - 4,880) | 1,420 (567 - 2,410) |
| LX | 2 | 5,940 (2,420 - 9,600) | 3,580 (1,450 - 4,870) | 509 (198 - 780) | 350 (136 - 487) | 2,240 (918 - 3,440) |
| BF | 2 | 5,180 (1,310 - 9,460) | 3,200 (1,090 - 4,840) | 215 (46.9 - 522) | 380 (161 - 490) | 628 (244 - 1,130) |
| LG | 2 | 5,240 (1,420 - 9,470) | 3,190 (1,090 - 4,810) | 232 (63.6 - 449) | 378 (160 - 489) | 868 (327 - 1,670) |
| PS | 1 | 272 (106 - 632) | 2,960 (717 - 4,790) | 115 (24.5 - 480) | 441 (296 - 495) | 442 (211 - 755) |
| CJ | 2 | 5,020 (1,040 - 9,460) | 2,630 (718 - 4,720) | 37 (5.5 - 203) | 408 (198 - 493) | 177 (63.7 - 379) |

Scenario - the optimal scenario for each local population (population expansion, recovery, and contraction are represented as 1, 2, and 3, respectively). *N*_anc_ - effective population size of ancestral population; *T*_anc_ - time before bottleneck (generations/years ago); *N*_bot_ - post-bottleneck effective population size; *T*_bot_ - time after bottleneck (generations/years ago); *N*_e_ - contemporary effective population size. The values in parentheses represented the 5% and 95% quantiles of each parameter. Site abbreviations correspond to those in Table 1 in the main text.

**Table S8** Bias and error estimates of posterior parameter distributions for the optimal scenario in each local population

| **(a) Mean Relative Bias** | | | | | | |
| --- | --- | --- | --- | --- | --- | --- |
| Sites | Parameter | *N*_anc_ | *T*_anc_ | *N*_bot_ | *T*_bot_ | *N*_e_ |
| CP | Mean | 0.192  (0.417) | 0.371  (0.250) | 2.759  (7.691) | 0.117  (-0.216) | 0.940  (6.819) |
|  | Median | 0.1808  (0.483) | 0.3826  (0.248) | 2.4181  (7.409) | 0.1849  (-0.216) | 0.8367  (6.180) |
|  | Mode | -0.1257 | 0.4545 | 1.5964 | 0.4435 | 0.6269 |
| BG | Mean | 0.238  (0.483) | 0.619  (0.338) | 18.315  (16.258) | -0.187  (-0.292) | 6.300  (17.896) |
|  | Median | 0.2288  (0.554) | 0.6774  (0.340) | 18.6764  (15.705) | -0.1484  (-0.290) | 5.6331  (16.387) |
|  | Mode | -0.1994 | 1.0615 | 20.6050 | -0.0042 | 5.2757 |
| GQ | Mean | 0.206  (0.394) | 0.277  (0.128) | 5.363  (7.732) | 0.045  (-0.193) | 3.136  (9.186) |
|  | Median | 0.1995  (0.461) | 0.3027  (0.127) | 5.4154  (7.427) | 0.0905  (-0.195) | 2.7971  (8.348) |
|  | Mode | -0.0414 | 0.4997 | 5.9012 | 0.2923 | 2.6294 |
| CS | Mean | 0.481  (0.431) | -0.274  (-0.167) | 1.001  (0.938) | 1.388  (3.391) | 3.278  (4.665) |
|  | Median | 0.4968  (0.433) | -0.3563  (-0.167) | 1.0756  (1.053) | 1.1643  (3.397) | 2.2742  (3.984) |
|  | Mode | 0.4314 | -0.4105 | 1.2940 | 0.9819 | 1.2242 |
| SN | Mean | 0.199  (0.359) | 0.258  (0.092) | 0.165  (1.342) | 0.160  (-0.173) | 0.221  (1.234) |
|  | Median | 0.1978  (0.422) | 0.2905  (0.094) | 0.1764  (1.263) | 0.2231  (-0.172) | 0.2298  (1.055) |
|  | Mode | 0.0870 | 0.6107 | 0.2202 | 0.5004 | 0.2629 |
| PJ | Mean | 0.174  (0.368) | 0.173  (0.017) | 0.127  (1.445) | 0.165  (-0.180) | 0.215  (1.884) |
|  | Median | 0.1681  (0.433) | 0.1970  (0.017) | 0.1199  (1.363) | 0.2265  (-0.179) | 0.2116  (1.651) |
|  | Mode | -0.1540 | 0.3756 | 0.1276 | 0.5010 | 0.2257 |
| XY | Mean | 0.158  (0.351) | 0.285  (0.108) | 0.197  (1.342) | 0.172  (-0.174) | 0.238  (1.952) |
|  | Median | 0.1467  (0.415) | 0.3167  (0.108) | 0.1802  (1.256) | 0.2300  (-0.177) | 0.2205  (1.718) |
|  | Mode | -0.1153 | 0.5880 | 0.1596 | 0.5514 | 0.1913 |
| WG | Mean | 0.180  (0.357) | 0.201  (0.039) | 0.093  (1.058) | 0.102  (-0.213) | 0.156  (1.308) |
|  | Median | 0.1748  (0.424) | 0.2344  (0.041) | 0.0905  (0.997) | 0.1581  (-0.216) | 0.1505  (1.124) |
|  | Mode | -0.0376 | 0.6206 | 0.0715 | 0.4643 | 0.1374 |
| JB | Mean | 0.138  (0.285) | 0.159  (-0.048) | 0.146  (0.461) | 0.150  (-0.155) | 0.194  (1.002) |
|  | Median | 0.1359  (0.345) | 0.1979  (-0.047) | 0.1496  (0.412) | 0.2010  (-0.155) | 0.1890  (0.841) |
|  | Mode | -0.0130 | 0.6057 | 0.2010 | 0.5472 | 0.1880 |
| QX | Mean | 0.168  (0.283) | 0.237  (0.002) | 0.233  (0.370) | 0.183  (-0.101) | 0.273  (0.820) |
|  | Median | 0.1733  (0.343) | 0.2957  (0.003) | 0.2390  (0.322) | 0.2304  (-0.103) | 0.2671  (0.669) |
|  | Mode | 0.1115 | 0.7129 | 0.3024 | 0.5454 | 0.2946 |
| LX | Mean | 0.147  (0.267) | 0.205  (0.031) | 0.129  (1.000) | 0.114  (-0.190) | 0.171  (0.731) |
|  | Median | 0.1472  (0.327) | 0.2345  (0.029) | 0.1456  (0.931) | 0.1687  (-0.191) | 0.1803  (0.592) |
|  | Mode | 0.0581 | 0.4801 | 0.2238 | 0.4809 | 0.2207 |
| BF | Mean | 0.182  (0.397) | 0.291  (0.168) | 0.289  (2.638) | 0.136  (-0.206) | 0.308  (3.464) |
|  | Median | 0.1707  (0.463) | 0.3022  (0.168) | 0.2202  (2.510) | 0.1966  (-0.203) | 0.2680  (3.095) |
|  | Mode | 0.0098 | 0.3329 | 0.0913 | 0.4896 | 0.2107 |
| LG | Mean | 0.177  (0.381) | 0.299  (0.132) | 0.230  (1.848) | 0.159  (-0.181) | 0.266  (2.459) |
|  | Median | 0.1714  (0.447) | 0.3257  (0.132) | 0.1996  (1.742) | 0.2215  (-0.181) | 0.2442  (2.181) |
|  | Mode | -0.1148 | 0.6553 | 0.1636 | 0.5350 | 0.2107 |
| PS | Mean | 0.212  (0.453) | 0.524  (0.304) | 14.984  (19.090) | -0.031  (-0.253) | 3.779  (13.294) |
|  | Median | 0.1975  (0.522) | 0.5713  (0.305) | 14.4355  (18.398) | 0.0173  (-0.253) | 3.3595  (12.144) |
|  | Mode | -0.1829 | 0.9199 | 13.5914 | 0.2513 | 2.9979 |
| CJ | Mean | 0.248  (0.484) | 0.380  (0.147) | 5.116  (3.930) | 0.533  (0.710) | 3.757  (9.906) |
|  | Median | 0.2373  (0.556) | 0.4250  (0.149) | 5.3309  (3.769) | 0.4515  (0.716) | 3.2711  (9.009) |
|  | Mode | -0.1125 | 0.7492 | 6.4208 | 0.1445 | 3.1068 |
|  |  |  |  |  |  |  |
| **(b) Median Relative Bias** | | | | | | |
| Sites | Parameter | *N*_anc_ | *T*_anc_ | *N*_bot_ | *T*_bot_ | *N*_e_ |
| CP | Mean | 0.009  (0.196) | 0.111  (7.473) | 1.042  (5.394) | -0.071  (-0.350) | 0.513  (5.672) |
|  | Median | -0.000  (0.252) | 0.126  (-0.007) | 0.835  (5.170) | -0.017  (-0.348) | 0.454  (5.119) |
|  | Mode | -0.271 | 0.143 | 0.405 | 0.203 | 0.261 |
| BG | Mean | 0.007  (0.203) | 0.220  (0.027) | 14.742  (14.266) | -0.265  (-0.385) | 5.186  (15.963) |
|  | Median | 0.000  (0.259) | 0.265  (0.029) | 14.656  (13.819) | -0.223  (-0.386) | 4.654  (14.657) |
|  | Mode | -0.390 | 0.525 | 13.545 | 0.059 | 4.138 |
| GQ | Mean | 0.032  (0.204) | 0.012  (-0.111) | 0.932  (5.203) | -0.138  (-0.358) | 0.597  (4.416) |
|  | Median | 0.022  (0.265) | 0.026  (-0.115) | 0.840  (4.999) | -0.081  (-0.361) | 0.524  (3.993) |
|  | Mode | -0.184 | 0.190 | 0.639 | 0.139 | 0.578 |
| CS | Mean | 0.228  (0.220) | -0.385  (-0.313) | 0.499  (0.367) | 0.884  (1.551) | 2.436  (3.386) |
|  | Median | 0.254  (0.230) | -0.464  (-0.313) | 0.567  (0.447) | 0.701  (1.557) | 1.771  (2.860) |
|  | Mode | 0.114 | -0.650 | 0.756 | 0.530 | 0.744 |
| SN | Mean | 0.024  (0.162) | -0.023  (-0.146) | -0.041  (0.886) | -0.065  (-0.335) | 0.006  (0.827) |
|  | Median | 0.026  (0.214) | -0.001  (-0.148) | -0.033  (0.821) | -0.016  (-0.335) | 0.015  (0.681) |
|  | Mode | -0.089 | 0.268 | 0.007 | 0.206 | 0.035 |
| PJ | Mean | 0.011  (0.177) | -0.067  (-0.194) | -0.063  (0.854) | -0.048  (-0.329) | 0.008  (1.318) |
|  | Median | 0.004  (0.228) | -0.049  (-0.193) | -0.067  (0.792) | -0.001  (-0.329) | 0.006  (1.140) |
|  | Mode | -0.271 | 0.109 | -0.051 | 0.225 | 0.011 |
| XY | Mean | -0.041  (0.125) | -0.025  (-0.165) | 0.011  (0.847) | -0.002  (-0.306) | 0.073  (1.540) |
|  | Median | -0.045  (0.181) | -0.004  (-0.168) | 0.001  (0.795) | 0.043  (-0.308) | 0.060  (1.343) |
|  | Mode | -0.260 | 0.241 | -0.005 | 0.308 | 0.009 |
| WG | Mean | 0.022  (0.180) | -0.038  (-0.170) | -0.056  (0.785) | -0.055  (-0.325) | -0.008  (1.049) |
|  | Median | 0.017  (0.234) | -0.013  (-0.170) | -0.059  (0.730) | -0.007  (-0.326) | -0.007  (0.880) |
|  | Mode | -0.185 | 0.327 | -0.063 | 0.256 | -0.022 |
| JB | Mean | 0.022  (0.162) | -0.076  (-0.241) | 0.001  (0.224) | -0.049  (-0.295) | 0.045  (0.721) |
|  | Median | 0.020  (0.215) | -0.045  (-0.239) | 0.005  (0.185) | -0.005  (-0.296) | 0.042  (0.578) |
|  | Mode | -0.125 | 0.276 | 0.039 | 0.301 | 0.038 |
| QX | Mean | 0.028  (0.126) | -0.021  (-0.212) | -0.004  (0.114) | -0.043  (-0.271) | 0.033  (0.490) |
|  | Median | 0.037  (0.184) | 0.023  (-0.213) | 0.002  (0.080) | -0.005  (-0.272) | 0.026  (0.364) |
|  | Mode | -0.032 | 0.340 | 0.056 | 0.256 | 0.053 |
| LX | Mean | 0.018  (0.121) | -0.023  (-0.172) | -0.042  (0.711) | -0.070  (-0.324) | -0.016  (0.457) |
|  | Median | 0.020  (0.172) | 0.004  (-0.175) | -0.028  (0.653) | -0.027  (-0.325) | 0.004  (0.344) |
|  | Mode | -0.065 | 0.199 | 0.050 | 0.226 | 0.060 |
| BF | Mean | -0.029  (0.153) | -0.017  (-0.118) | 0.015  (1.865) | -0.062  (-0.345) | 0.076  (2.825) |
|  | Median | -0.036  (0.205) | -0.017  (-0.119) | -0.043  (1.769) | -0.012  (-0.342) | 0.041  (2.492) |
|  | Mode | -0.172 | 0.016 | -0.144 | 0.226 | 0.008 |
| LG | Mean | -0.006  (0.171) | -0.012  (-0.144) | 0.030  (1.139) | -0.047  (-0.327) | 0.071  (1.800) |
|  | Median | -0.009  (0.228) | 0.014  (-0.139) | 0.006  (1.069) | 0.003  (-0.332) | 0.055  (1.579) |
|  | Mode | -0.258 | 0.281 | -0.024 | 0.275 | 0.026 |
| PS | Mean | 0.019  (0.218) | 0.194  (0.029) | 11.802  (17.743) | -0.154  (-0.368) | 2.347  (11.287) |
|  | Median | 0.010  (0.274) | 0.241  (0.030) | 9.924  (16.980) | -0.106  (-0.371) | 2.115  (10.353) |
|  | Mode | -0.336 | 0.493 | 5.917 | 0.152 | 1.456 |
| CJ | Mean | 0.064  (0.248) | 0.104  (-0.066) | 1.253  (1.051) | -0.291  (-0.316) | 1.691  (5.521) |
|  | Median | 0.054  (0.306) | 0.141  (-0.060) | 1.264  (0.995) | -0.259  (-0.310) | 1.415  (4.959) |
|  | Mode | -0.277 | 0.340 | 1.415 | -0.251 | 1.322 |
|  |  |  |  |  |  |  |
| **(c) Square Root of Mean Square Error** | | | | | | |
| Sites | Parameter | *N*_anc_ | *T*_anc_ | *N*_bot_ | *T*_bot_ | *N*_e_ |
| CP | Mean | 0.586  (0.778) | 0.948  (0.829) | 5.929  (10.354) | 0.815  (0.606) | 1.894  (8.382) |
|  | Median | 0.577  (0.840) | 0.961  (0.826) | 5.509  (9.993) | 0.876  (0.603) | 1.756  (7.631) |
|  | Mode | 0.503 | 1.131 | 4.492 | 1.116 | 1.526 |
| BG | Mean | 0.647  (0.867) | 1.278  (0.985) | 23.269  (19.072) | 0.431  (0.400) | 7.994  (20.351) |
|  | Median | 0.639  (0.936) | 1.338  (0.988) | 24.317  (18.426) | 0.456  (0.400) | 7.152  (18.649) |
|  | Mode | 0.631 | 1.778 | 29.265 | 0.757 | 7.023 |
| GQ | Mean | 0.596  (0.765) | 0.840  (0.739) | 11.231  (11.009) | 0.731  (0.580) | 7.225  (15.852) |
|  | Median | 0.590  (0.827) | 0.856  (0.738) | 11.712  (10.599) | 0.776  (0.582) | 6.362  (14.518) |
|  | Mode | 0.601 | 1.017 | 14.117 | 1.101 | 6.001 |
| CS | Mean | 0.851  (0.798) | 0.582  (0.506) | 1.847  (1.922) | 2.318  (5.803) | 5.857  (6.288) |
|  | Median | 0.872  (0.800) | 0.651  (0.504) | 1.920  (2.065) | 2.072  (5.818) | 3.155  (5.435) |
|  | Mode | 1.055 | 0.891 | 2.216 | 1.940 | 2.089 |
| SN | Mean | 0.565  (0.698) | 0.834  (0.692) | 0.742  (1.990) | 0.715  (0.525) | 0.787  (1.858) |
|  | Median | 0.563  (0.756) | 0.864  (0.693) | 0.754  (1.899) | 0.769  (0.526) | 0.801  (1.658) |
|  | Mode | 0.583 | 1.201 | 0.813 | 1.032 | 0.877 |
| PJ | Mean | 0.563  (0.726) | 0.714  (0.610) | 0.659  (2.324) | 0.706  (0.510) | 0.742  (2.677) |
|  | Median | 0.558  (0.786) | 0.732  (0.612) | 0.655  (2.227) | 0.757  (0.511) | 0.740  (2.406) |
|  | Mode | 0.437 | 0.907 | 0.665 | 1.011 | 0.762 |
| XY | Mean | 0.556  (0.716) | 0.962  (0.802) | 0.799  (2.613) | 0.665  (0.481) | 0.707  (2.580) |
|  | Median | 0.548  (0.775) | 0.994  (0.800) | 0.788  (2.500) | 0.712  (0.479) | 0.690  (2.312) |
|  | Mode | 0.482 | 1.276 | 0.773 | 1.020 | 0.676 |
| WG | Mean | 0.545  (0.690) | 0.729  (0.619) | 0.564  (1.515) | 0.514  (0.419) | 0.609  (1.722) |
|  | Median | 0.541  (0.750) | 0.755  (0.621) | 0.566  (1.450) | 0.552  (0.419) | 0.609  (1.523) |
|  | Mode | 0.491 | 1.135 | 0.563 | 0.816 | 0.599 |
| JB | Mean | 0.485  (0.594) | 0.676  (0.547) | 0.603  (1.075) | 0.629  (0.470) | 0.653  (1.492) |
|  | Median | 0.484  (0.646) | 0.706  (0.548) | 0.607  (1.029) | 0.668  (0.470) | 0.649  (1.320) |
|  | Mode | 0.455 | 1.106 | 0.648 | 0.995 | 0.656 |
| QX | Mean | 0.510  (0.598) | 0.786  (0.604) | 0.847  (0.978) | 0.800  (0.603) | 0.887  (1.455) |
|  | Median | 0.514  (0.649) | 0.838  (0.605) | 0.853  (0.927) | 0.843  (0.600) | 0.883  (1.292) |
|  | Mode | 0.534 | 1.249 | 0.933 | 1.137 | 0.930 |
| LX | Mean | 0.488  (0.581) | 0.718  (0.591) | 0.560  (1.398) | 0.535  (0.426) | 0.598  (1.163) |
|  | Median | 0.489  (0.632) | 0.743  (0.590) | 0.571  (1.332) | 0.572  (0.426) | 0.602  (1.020) |
|  | Mode | 0.489 | 1.032 | 0.637 | 0.849 | 0.631 |
| BF | Mean | 0.591  (0.773) | 0.921  (0.800) | 1.004  (3.940) | 0.620  (0.472) | 0.912  (4.335) |
|  | Median | 0.582  (0.834) | 0.933  (0.801) | 0.954  (3.781) | 0.665  (0.472) | 0.875  (3.907) |
|  | Mode | 0.499 | 1.035 | 0.905 | 0.937 | 0.823 |
| LG | Mean | 0.570  (0.741) | 0.938  (0.787) | 0.855  (3.119) | 0.707  (0.520) | 0.862  (3.277) |
|  | Median | 0.566  (0.802) | 0.964  (0.787) | 0.835  (2.978) | 0.758  (0.520) | 0.850  (2.951) |
|  | Mode | 0.478 | 1.335 | 0.813 | 1.057 | 0.817 |
| PS | Mean | 0.611  (0.824) | 1.152  (0.925) | 20.265  (21.155) | 0.503  (0.473) | 6.308  (16.487) |
|  | Median | 0.599  (0.891) | 1.205  (0.927) | 20.234  (20.397) | 0.528  (0.473) | 5.601  (15.107) |
|  | Mode | 0.556 | 1.633 | 21.937 | 0.736 | 5.316 |
| CJ | Mean | 0.635  (0.850) | 0.967  (0.750) | 11.462  (8.588) | 2.489  (3.094) | 7.087  (15.724) |
|  | Median | 0.627  (0.920) | 1.014  (0.750) | 12.088  (8.288) | 2.290  (3.105) | 6.237  (14.382) |
|  | Mode | 0.622 | 1.390 | 15.026 | 2.648 | 5.954 |

Mean (a) and median (b) relative bias as well as the square root of mean square error (c) are given. Values in parentheses are calculated for parameters estimated using the prior distribution. *N*_anc_ - effective population size of ancestral population; *T*_anc_ - time before bottleneck (generations/years ago); *N*_bot_ - post-bottleneck effective population size; *T*_bot_ - time after bottleneck (generations/years ago); *N*_e_ - contemporary effective population size. Site abbreviations correspond to those in Table 1 in the main text.

**Table S9** Pairwise *F*_ST_ calculated by the 71,910 SNP loci (below diagonal) and the 13 SSR loci (above diagonal) between local populations of *P. plancyi*

|  |  | West | | | | | |  | East | | | | | | |  | Island | |
| --- | --- | --- | --- | --- | --- | --- | --- | --- | --- | --- | --- | --- | --- | --- | --- | --- | --- | --- |
|  |  | QX | JB | CS | SN | BG | GQ |  | CP | PJ | LX | WG | XY | BF | LG |  | PS | CJ |
| West | QX | - | 0.011 | 0.024 | 0.005 | 0.026 | 0.055 |  | 0.043 | -0.001 | 0.003 | 0.010 | 0.009 | 0.018 | 0.015 |  | 0.040 | 0.070 |
|  | JB | 0.005 | - | 0.017 | 0.001 | 0.034 | 0.058 |  | 0.036 | 0.015 | 0.009 | 0.009 | 0.014 | 0.021 | 0.014 |  | 0.031 | 0.048 |
|  | CS | 0.041 | 0.044 | - | 0.029 | 0.058 | 0.078 |  | 0.063 | 0.042 | 0.025 | 0.025 | 0.023 | 0.040 | 0.044 |  | 0.049 | 0.068 |
|  | SN | -0.002 | 0.005 | 0.041 | - | 0.027 | 0.055 |  | 0.046 | 0.006 | 0.010 | 0.011 | 0.016 | 0.016 | 0.007 |  | 0.037 | 0.062 |
|  | BG | 0.030 | 0.033 | 0.068 | 0.030 | - | 0.058 |  | 0.044 | 0.017 | 0.023 | 0.031 | 0.039 | 0.034 | 0.033 |  | 0.050 | 0.069 |
|  | GQ | 0.070 | 0.068 | 0.107 | 0.071 | 0.086 | - |  | 0.055 | 0.045 | 0.049 | 0.046 | 0.059 | 0.060 | 0.064 |  | 0.049 | 0.070 |
|  |  |  |  |  |  |  |  |  |  |  |  |  |  |  |  |  |  |  |
| East | CP | 0.039 | 0.038 | 0.076 | 0.039 | 0.053 | 0.065 |  | - | 0.033 | 0.031 | 0.024 | 0.043 | 0.030 | 0.047 |  | 0.039 | 0.076 |
|  | PJ | 0.007 | 0.010 | 0.047 | 0.000 | 0.034 | 0.076 |  | 0.041 | - | 0.002 | 0.011 | 0.014 | 0.006 | 0.004 |  | 0.034 | 0.063 |
|  | LX | -0.002 | 0.005 | 0.040 | -0.001 | 0.029 | 0.071 |  | 0.039 | 0.006 | - | 0.007 | 0.009 | 0.009 | 0.013 |  | 0.039 | 0.055 |
|  | WG | 0.011 | 0.012 | 0.050 | 0.010 | 0.036 | 0.067 |  | 0.035 | 0.012 | 0.011 | - | 0.006 | 0.013 | 0.019 |  | 0.032 | 0.064 |
|  | XY | 0.003 | 0.007 | 0.043 | 0.003 | 0.032 | 0.073 |  | 0.040 | 0.005 | 0.003 | 0.007 | - | 0.011 | 0.011 |  | 0.034 | 0.055 |
|  | BF | 0.009 | 0.014 | 0.049 | 0.009 | 0.038 | 0.078 |  | 0.046 | 0.011 | 0.009 | 0.016 | 0.006 | - | 0.018 |  | 0.027 | 0.070 |
|  | LG | 0.005 | 0.010 | 0.044 | 0.004 | 0.033 | 0.073 |  | 0.038 | 0.005 | 0.004 | 0.009 | 0.003 | 0.009 | - |  | 0.044 | 0.060 |
|  |  |  |  |  |  |  |  |  |  |  |  |  |  |  |  |  |  |  |
| Island | PS | 0.036 | 0.032 | 0.074 | 0.036 | 0.054 | 0.060 |  | 0.042 | 0.041 | 0.037 | 0.031 | 0.038 | 0.044 | 0.038 |  | - | 0.045 |
|  | CJ | 0.057 | 0.053 | 0.095 | 0.057 | 0.078 | 0.088 |  | 0.068 | 0.061 | 0.058 | 0.026 | 0.056 | 0.065 | 0.061 |  | 0.047 | - |

For easily visualizing the intensities of isolation effects caused by the Huangpu River and islands, the sampling sites are categorized into three groups based on the geographic factors: (a) west side of Huangpu river, (b) east side of Huangpu river, and (c) islands. Site abbreviations correspond to those in Table 1 in the main text.

**Table S10** Recent migration rates between each pair of genetic clusters of *P. plancyi* estimated from the 1,611 SNP loci using BayesAss3

|  | Peripheral | CP | BG | GQ | CS | WG | PS | CJ |
| --- | --- | --- | --- | --- | --- | --- | --- | --- |
| Peripheral | 0.970 | 0.004 | 0.004 | 0.006 | 0.004 | 0.004 | 0.005 | 0.004 |
| CP | 0.167 | 0.685 | 0.018 | 0.052 | 0.019 | 0.019 | 0.023 | 0.018 |
| BG | 0.204 | 0.018 | 0.685 | 0.019 | 0.018 | 0.018 | 0.019 | 0.019 |
| GQ | 0.037 | 0.019 | 0.019 | 0.833 | 0.019 | 0.018 | 0.019 | 0.037 |
| CS | 0.204 | 0.019 | 0.019 | 0.018 | 0.685 | 0.018 | 0.019 | 0.019 |
| WG | 0.204 | 0.019 | 0.019 | 0.019 | 0.018 | 0.685 | 0.018 | 0.019 |
| PS | 0.165 | 0.018 | 0.018 | 0.029 | 0.019 | 0.019 | 0.696 | 0.036 |
| CJ | 0.074 | 0.019 | 0.018 | 0.018 | 0.019 | 0.019 | 0.037 | 0.796 |

The value in row *i*, column *j* represents the fraction of individuals in the cluster *i* that are migrants derived from the cluster *j* (per generation). Peripheral represents the genetic cluster which contains local populations of QX, JB, SN, PJ, LX, XY, BF, and LG according to the population structure analysis results. The clusters containing single local population use the same abbreviations with the local populations corresponding to those in Table 1 in the main text.

**Table S11** Recent migration rates between each pair of genetic clusters of *P. plancyi* estimated from the 13 SSR loci using BayesAss3

|  | Peripheral | CP | BG | GQ | CS | WG | PS | CJ |
| --- | --- | --- | --- | --- | --- | --- | --- | --- |
| Peripheral | 0.854 | 0.021 | 0.021 | 0.021 | 0.021 | 0.021 | 0.021 | 0.021 |
| CP | 0.074 | 0.704 | 0.037 | 0.037 | 0.037 | 0.037 | 0.037 | 0.037 |
| BG | 0.074 | 0.037 | 0.704 | 0.037 | 0.037 | 0.037 | 0.037 | 0.037 |
| GQ | 0.074 | 0.037 | 0.037 | 0.704 | 0.037 | 0.037 | 0.038 | 0.037 |
| CS | 0.074 | 0.037 | 0.037 | 0.037 | 0.704 | 0.037 | 0.037 | 0.037 |
| WG | 0.074 | 0.037 | 0.037 | 0.037 | 0.037 | 0.704 | 0.037 | 0.037 |
| PS | 0.074 | 0.037 | 0.037 | 0.037 | 0.037 | 0.037 | 0.704 | 0.037 |
| CJ | 0.074 | 0.037 | 0.037 | 0.037 | 0.037 | 0.037 | 0.037 | 0.704 |

The value in row *i*, column *j* represents the fraction of individuals in the cluster *i* that are migrants derived from the cluster *j* (per generation). Peripheral represents the genetic cluster which contains local populations of QX, JB, SN, PJ, LX, XY, BF, and LG according to the population structure analysis results. The clusters containing single local population use the same abbreviations with the local populations corresponding to those in Table 1 in the main text.

**FIGURE LEGENDS**

**Figure S1** Model checking plots of PCA scores for the 15 local populations. Small open dots with coloration represent prior simulations, while the larger filled dots correspond to a dataset simulated with parameters drawn from the posterior distributions (1,000 datasets are randomly shown here). The colors of these dots represent optimal scenarios in different local populations (red - population recovery; green - population expansion). The large yellow dot corresponds to the real datasets for each local population. Site abbreviations correspond to those in Table 1 in the main text.

**Figure S2** Network of 68 *Cyt-b* gene haplotypes of *P.* *plancyi*. The sizes of the circles represent the number of individuals sharing the haplotypes. The distances between circles show the variation between two haplotypes (e.g., 1-bp difference between H3 and H66).

**Figure S3** Maximum likelihood tree constructed based on the *Cyt-b* gene data. The numbers to the left of the branches show the bootstrap proportions > 75%. The name of each haplotype contains: (1) the ID number of the haplotype, which was as same as those in the haplotype network of Figure S2; (2) the number of individuals with the haplotype; and (3) the abbreviations of the local populations where the haplotype appeared. Site abbreviations correspond to those in Table 1 in the main text.

**Figure S4** Bayesian tree constructed based on the *Cyt-b* gene data. The numbers to the left of branches are Bayesian posterior probabilities > 95%. The name of each haplotype contains: (1) the ID number of the haplotype, which was as same as those in the haplotype network of Figure S2; (2) the number of individuals with the haplotype; and (3) the abbreviations of the local populations where the haplotype appeared. Site abbreviations correspond to those in Table 1 in the main text.

**Figure S5** Estimations of *ΔK* for (a) SSRs and (b) SNPs in STRUCTURE analysis. The optimal number of clusters appeared when *K* = 2 and *K* = 4 for SSRs and when *K* = 2, *K* = 5, and *K* = 8 for SNPs.

**Figure S6** Number of principal components retained for the DAPC analysis of SSRs. The function of *a.*score in the R package *adegenet* identified *n* = 39 as the optimal number PCs to retain.

**Figure S7** Cross-validation errors from Admixture for *K* from 1 to 15.

**Figure S8** Bar plots of population structure analyses using Admixture. All plots with *K* from 2 to 8 are shown. Site abbreviations correspond to those in Table 1 in the main text.

**Figure S9** Number of principal components retained for the DAPC analysis of SNPs. The function of *a.*score in the R package *adegenet* identified *n* = 23 as the optimal number of PCs to retain.

**Figure S10** Plot of the MCMC chain convergence calculated in BayesAss by SSR data.

**FIGURES**

**Figure S1**


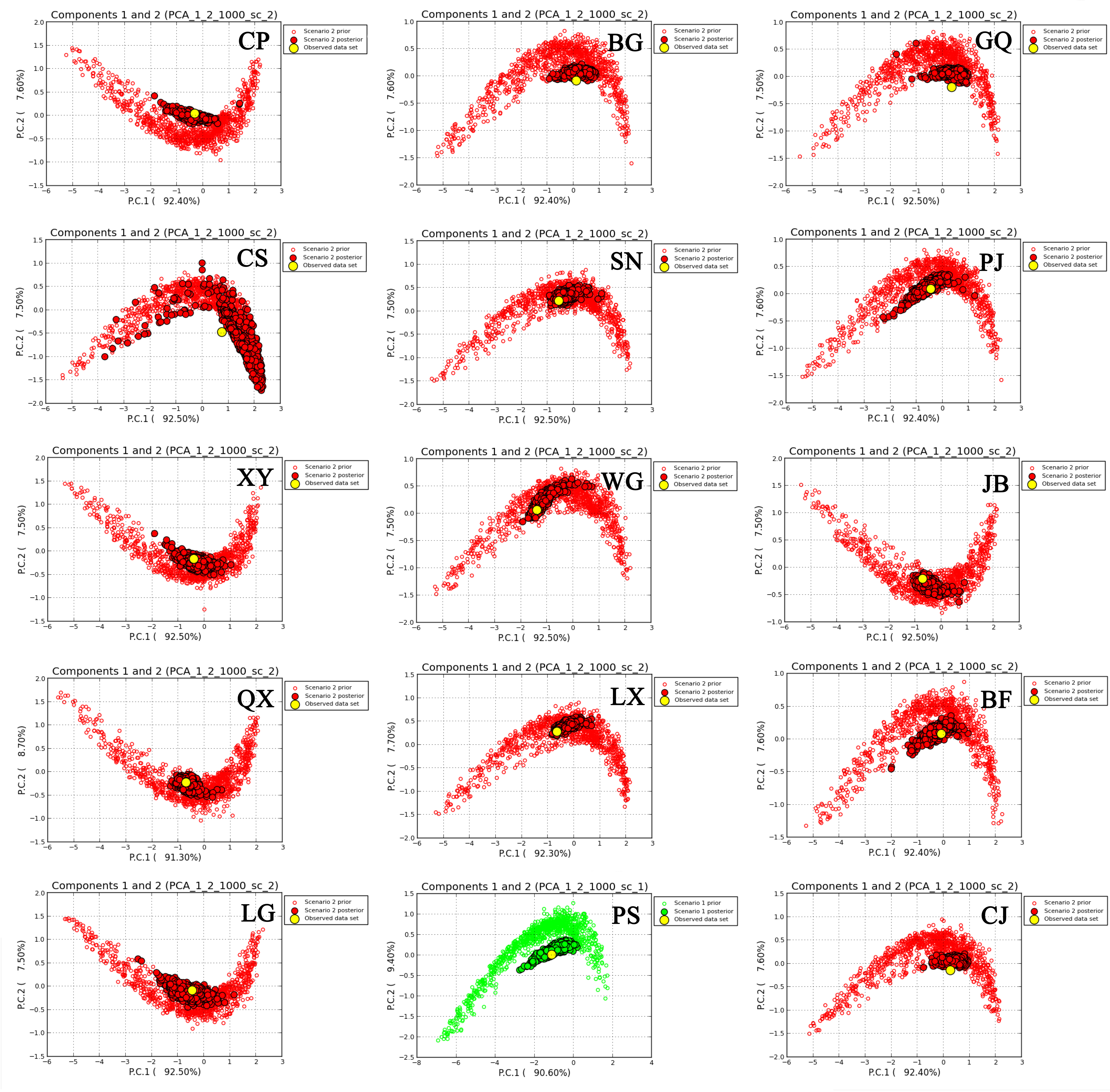


**Figure S2**


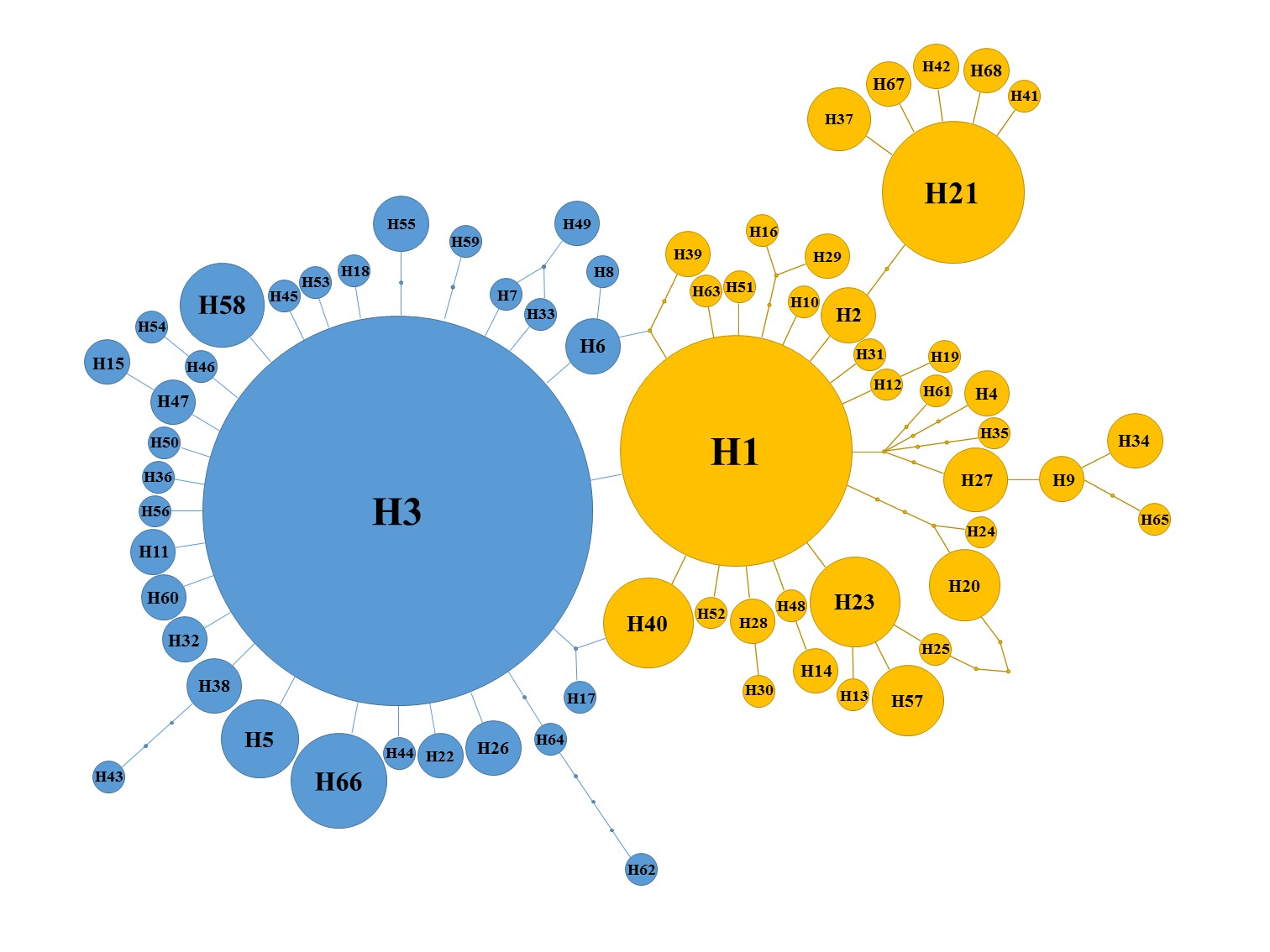


**Figure S3**


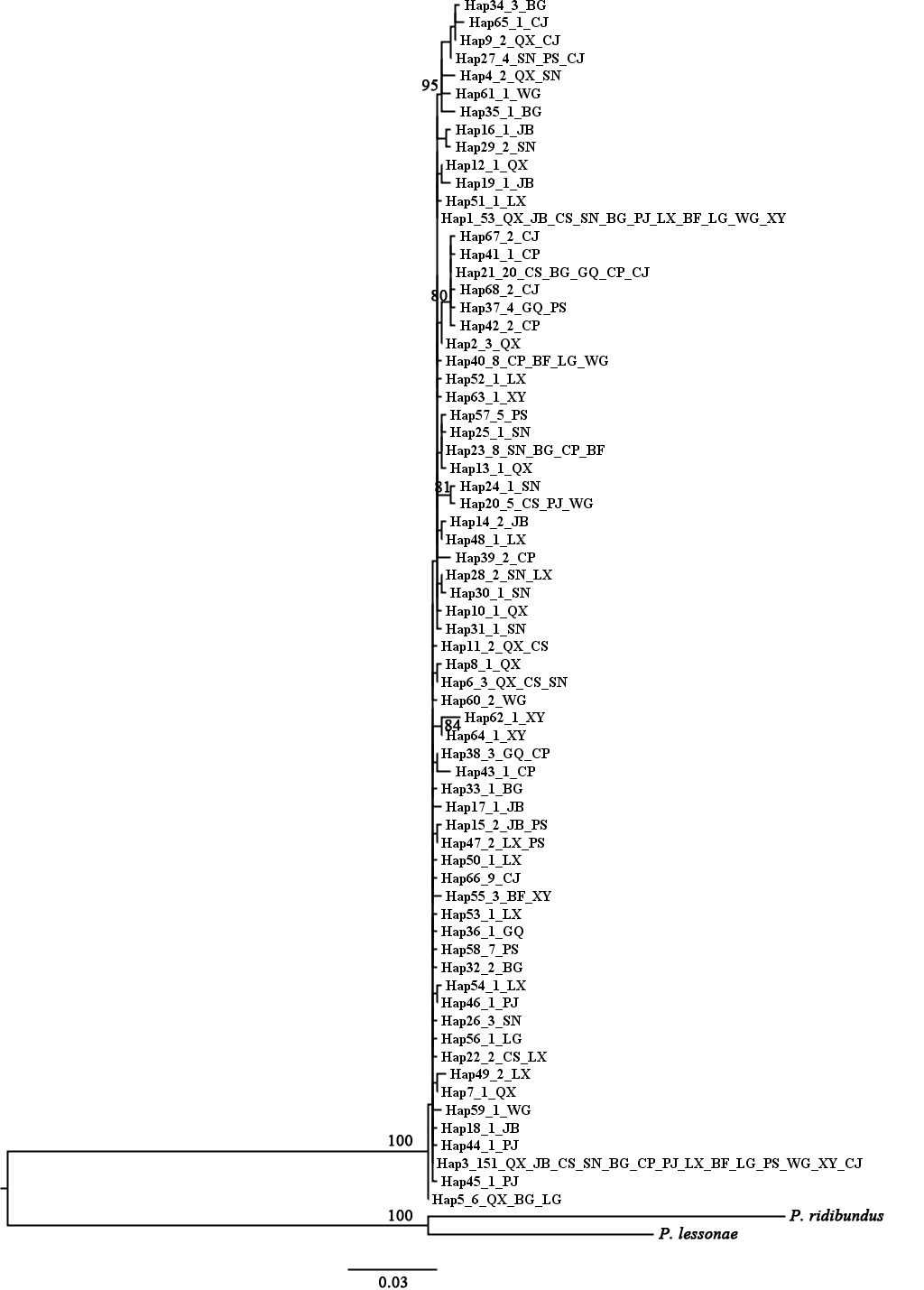


**Figure S4**


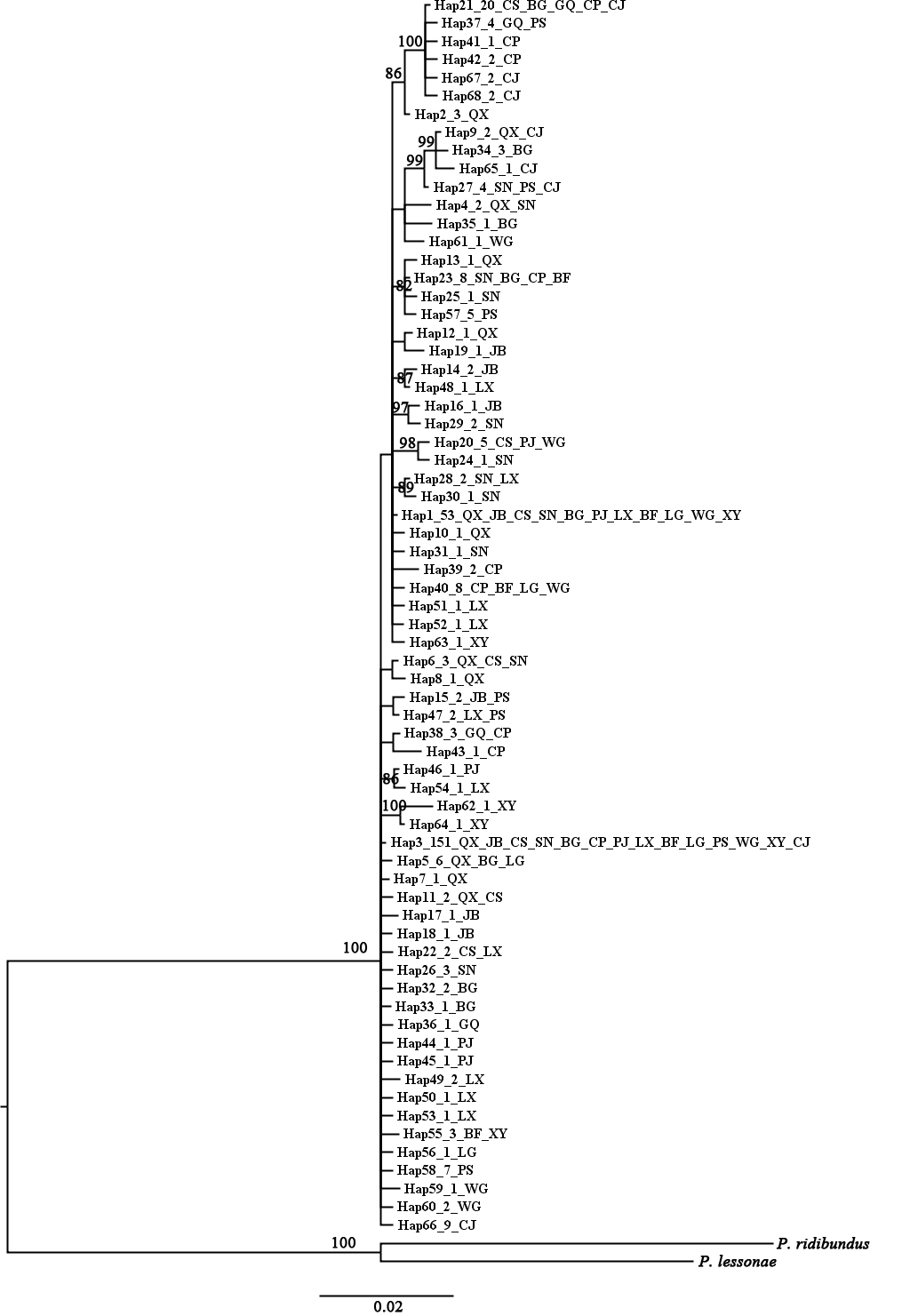


**Figure S5**


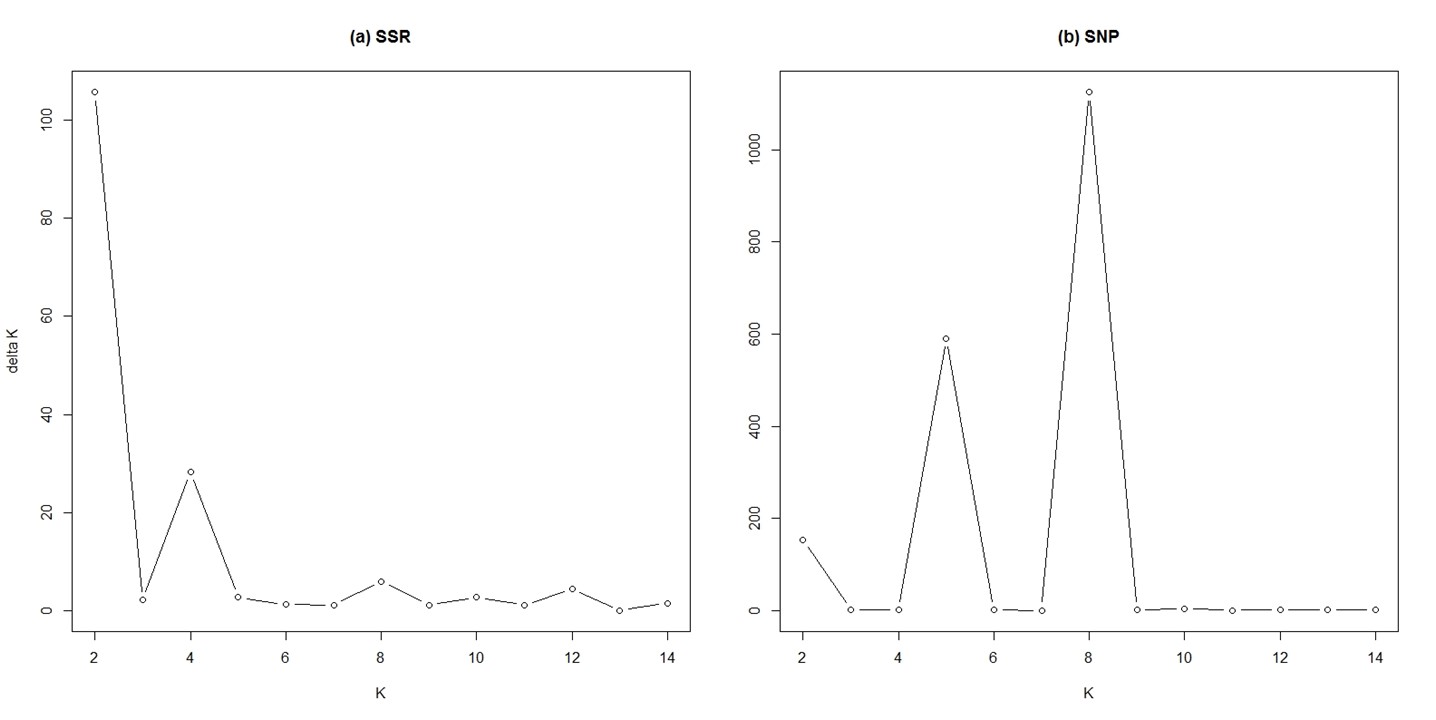


**Figure S6**


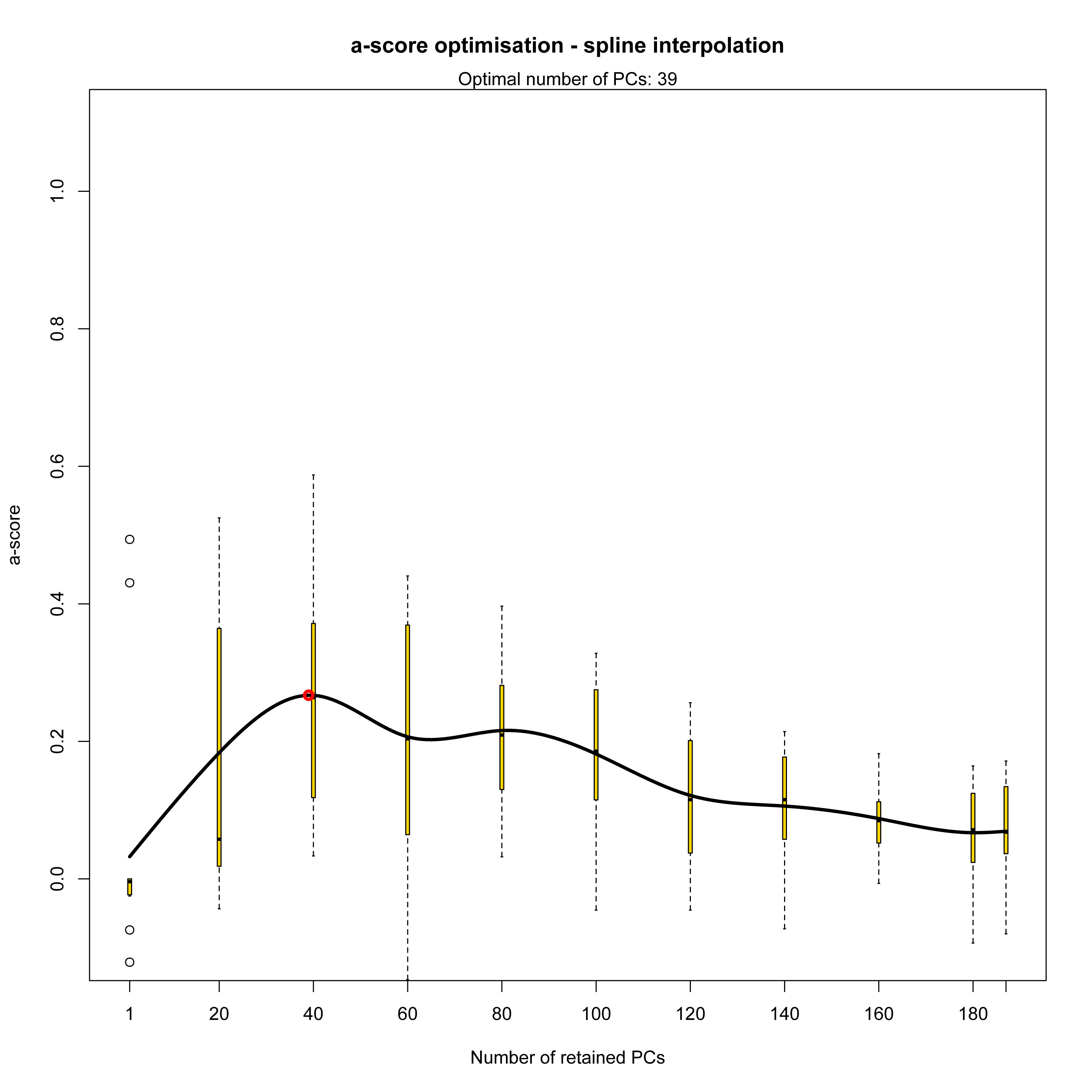


**Figure S7**


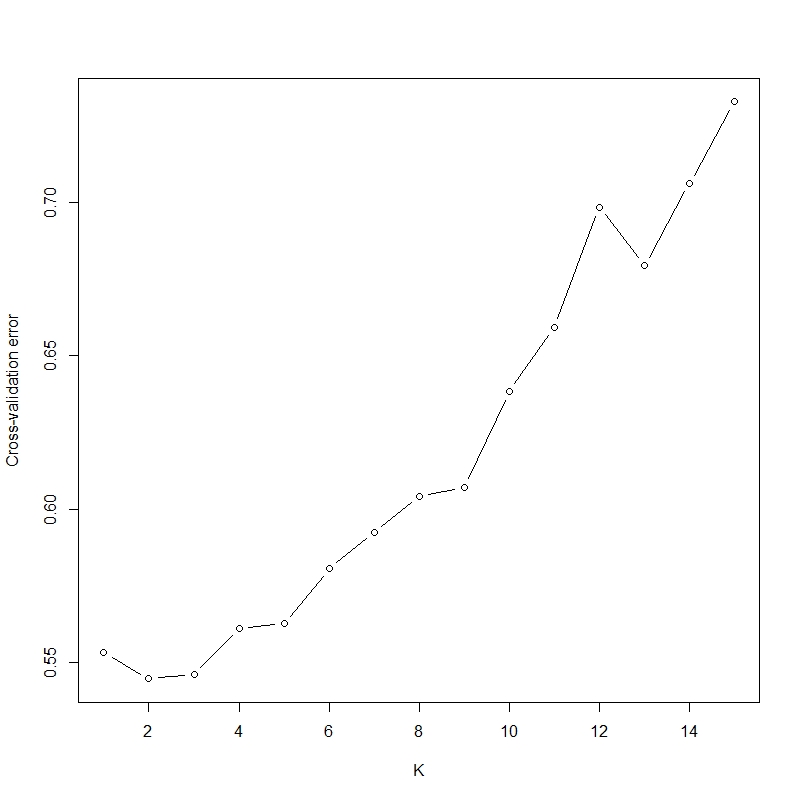


**Figure S8**


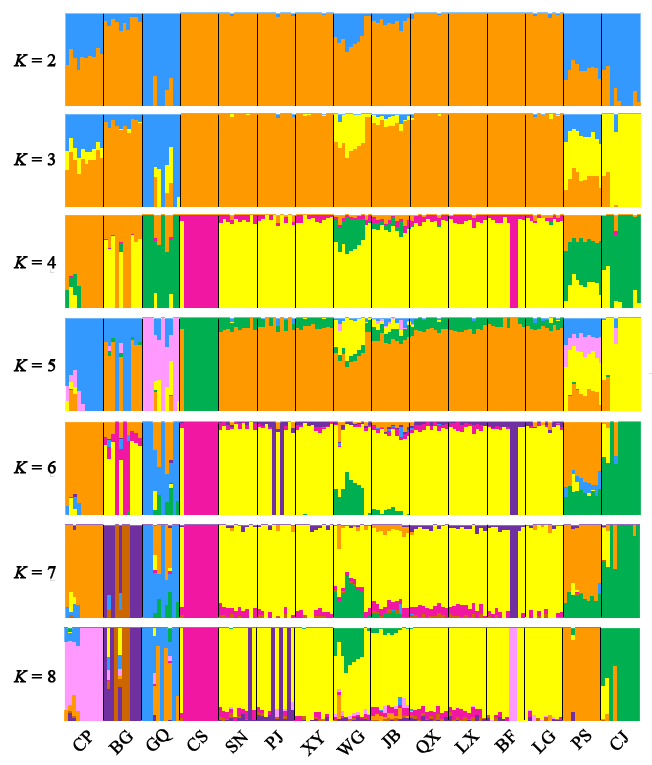


**Figure S9**


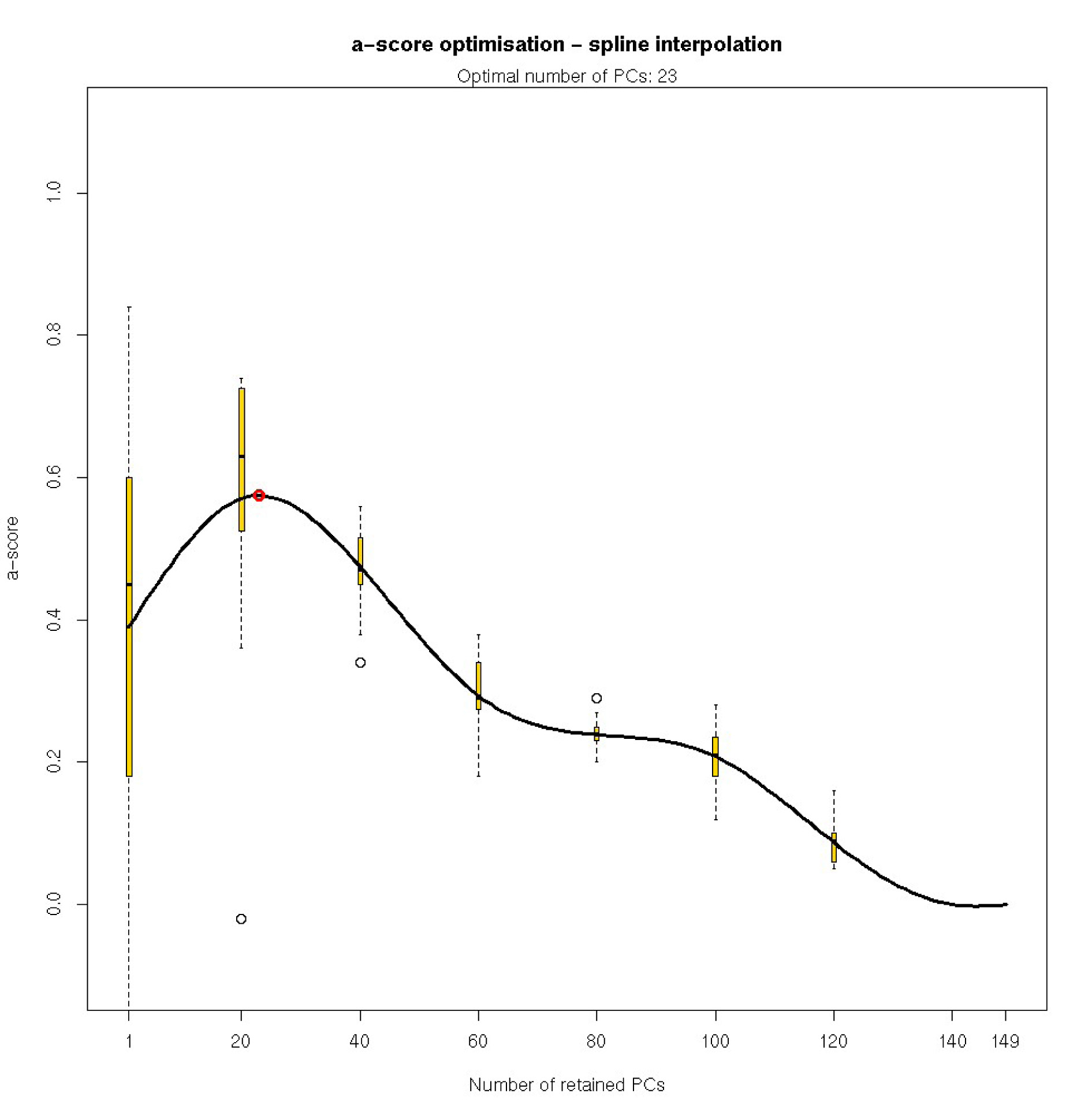


**Figure S10**


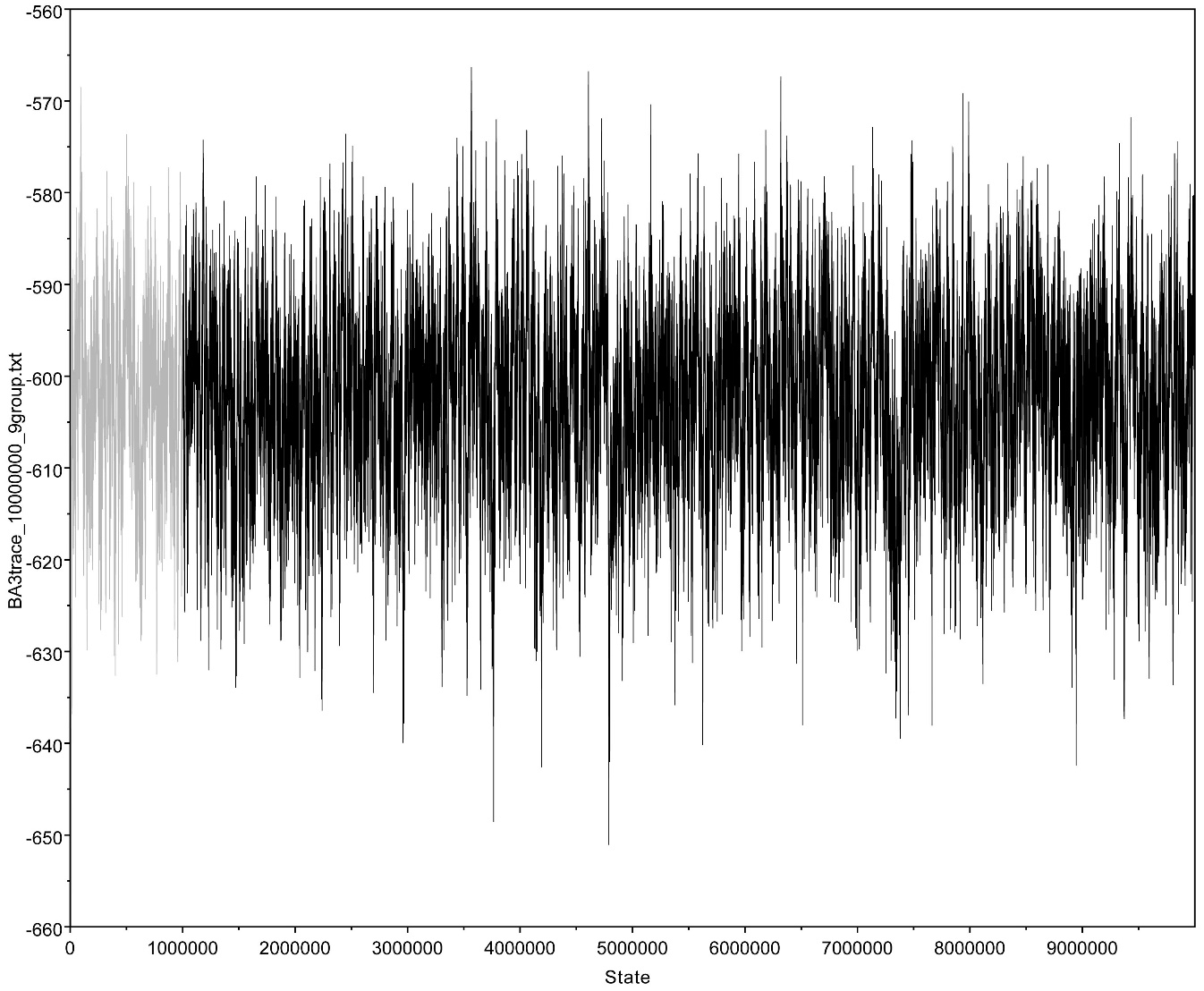


**Literature Cited**

Dai, J., Zhou, K. (2009). Development of microsatellite loci for *Pelophylax plancyi* and cross-amplification in other ranid species. *Conservation Genetics*, *10*(3), 763-766.

Du, J., Yan, J., Zhou, K. (2012). Isolation of microsatellite markers for *Pelophylax nigromaculata* and a tentative application in detecting interspecific introgression. *Gene*, *508*(1), 130-134.

Fu, L., Niu, B., Zhu, Z., Wu, S., Li, W. (2012). CD-HIT: accelerated for clustering the next-generation sequencing data. *Bioinformatics*, *28*(23), 3150-3152.

Magoc, T., Salzberg, S.L. (2011). FLASH: fast length adjustment of short reads to improve genome assemblies. *Bioinformatics*, *27*(21), 2957-2963.

Thiel, T., Michalek, W., Varshney, R., Graner, A. (2003). Exploiting EST databases for the development and characterization of gene-derived SSR-markers in barley (*Hordeum vulgare L.*). *Theoretical and Applied Genetics*, *106*(3), 411-422.
